# Genomic insights into adaptation and inbreeding among Sub-Saharan African cattle from pastoral and agropastoral systems

**DOI:** 10.3389/fgene.2024.1430291

**Published:** 2024-07-25

**Authors:** Oludayo M. Akinsola, Abdulraheem A. Musa, Lal Muansangi, Sanchit P. Singh, Sabyasachi Mukherjee, Anupama Mukherjee

**Affiliations:** ^1^ Department of Theriogenology and Production, Faculty of Veterinary Medicine, University of Jos, Jos, Nigeria; ^2^ Research Institute for Farm Animal Biology (FBN), Dummerstorf, Germany; ^3^ Animal Genetics and Breeding Division, Indian Council of Agricultural Research (ICAR)-National Dairy Research Institute (NDRI), Karnal, Haryana, India

**Keywords:** runs of homozygosity, integrated haplotype score, cattle genetic adaptation, inbreeding effects, tropical livestock genetics, disease resistance in cattle, environmental stress adaptability

## Abstract

**Background:**

In Sub-Saharan Africa (SSA), cattle are crucial for socioeconomic stability yet face numerous environmental stressors such as diseases, parasites, and extreme heat within pastoral and agropastoral systems. Despite their significance, gaps remain in understanding how genetic diversity and inbreeding influence traits essential for disease resistance and environmental adaptability. This study examines the genomic adaptations that enable SSA cattle to thrive under these conditions and assesses the impact of inbreeding on such adaptive traits.

**Methods:**

We analyzed genomic data from 113 cattle across four breeds—Kuri, N’dama, Zebu-Fulani, and Zebu-Bororo—employing Runs of Homozygosity (ROH) and Integrated Haplotype Score (iHS) analyses to identify historical and recent genetic selections. Strict quality controls using PLINK software ensured accurate genomic pattern identification related to adaptation and inbreeding.

**Results:**

ROH analysis revealed islands with genes such as *RSAD2, CMPK2,* and *NOTCH1*, which are involved in immune response and cellular stress management, highlighting regions of historical selection that have likely provided adaptive advantages in overcoming environmental and pathogenic stresses. In contrast, iHS analysis identified genes under recent selection like *HIPK1*, involved in stress response regulation, and *EPHA5*, which plays a crucial role in neural development and synaptic functions, potentially equipping these breeds with novel adaptations to ongoing and emergent environmental challenges.

**Conclusion:**

This research confirms that selective pressures inherent in pastoral and agropastoral systems profoundly influence the genetic structure of SSA cattle. By delineating the genetic bases of key adaptive traits, our study offers crucial insights for targeted breeding programs to enhance cattle resilience and productivity. These findings provide a valuable framework for future genetic improvements and conservation strategies, crucial for sustainable livestock management and economic stability in SSA.

## 1 Introduction

Sub-Saharan Africa (SSA) is home to diverse cattle production systems that are foundational to the livelihoods, culture, and food security of millions. Among these systems, pastoral and agropastoral systems are predominant and adaptive livestock management practices that have profoundly influenced the genetic architecture of indigenous cattle breeds over centuries through environmental and human-mediated selection pressures (Kim et al., 2020; Xia et al., 2023). These systems are characterized by seasonal and transhumant movements of livestock, responding to climatic variability and pasture availability while also significantly shaping the socioeconomic and cultural fabric of the region (Behnke et al., 2011).

The genetic diversity in SSA cattle, as represented by breeds such as the Kuri, N’dama, Zebu-Bororo, and Zebu-Fulani, exhibits a complex mosaic of traits evolved under a broad spectrum of environmental pressures. These breeds, shaped by both natural and anthropogenic selection pressures, demonstrate remarkable resilience to local stressors like diseases, parasites, and heat stress—traits that are less commonly observed in temperate and commercially improved breeds, which are often selected primarily for high productivity traits such as milk yield and growth rates (Orenge et al., 2012; Taye et al., 2017).

A central aspect of understanding the resilience and adaptability of these cattle breeds is the analysis of genetic phenomena such as runs of homozygosity (ROH) and selective sweeps. ROH, indicative of historical inbreeding and genetic drift, helps reveal genetic diversity patterns crucial for understanding the adaptive potential of cattle populations. Extensive studies in temperate and improved breeds have analyzed ROH, linking them to beneficial and deleterious traits. For example, a previous study investigated ROH patterns across cattle types and climatic zones, identifying genes associated with environmental adaptation, disease resistance, coat color, and production traits (Falchi et al., 2023). Similarly, another study identified temperament and body size genes crucial for stress responsiveness and climate tolerance in Brazilian cattle breeds (Peripolli et al., 2020).

Recent research has utilized various statistical methods and advanced genomic tools such as the integrated haplotype score (iHS) to identify candidate genes associated with traits like trypanotolerance in the Sheko breed, revealing genes such as *MIGA1, SPAG11B, ERN1,* and *CAPG* linked to anemia, immune response, and neurological functions (Mekonnen et al., 2019). Another study also highlighted the need for further functional investigations after identifying a prominent selection signature on BTA23 in the Muturu breed (Tijjani et al., 2019).

Despite the vital role of SSA cattle in their ecosystems and economies, there remains a notable deficiency in comprehensive genomic studies focused on these breeds. Research on genome-wide ROH and the analysis of selective sweeps is limited, and comparative studies on different genomic measures of inbreeding across various indigenous breeds within the region are lacking. This gap in research hinders a full understanding of how breeding and selection pressures have shaped these cattle’s genetic diversity and adaptive capacities, which are crucial for developing effective breeding strategies and conservation efforts.

This study aims to bridge these gaps by (i) characterizing the genomic diversity of SSA cattle through detailed analysis of ROH and assessing the impact of various genomic measures of inbreeding across different breeds and (ii) analyzing selective sweeps using ROH islands and iHS to identify genomic regions under selection that contribute to the adaptive traits of SSA cattle, thereby enhancing our understanding of their genetic resilience and adaptability.

## 2 Materials and methods

### 2.1 Animals

The genetic data for this study was sourced from the Web-Interfaced Next-generation Database dedicated to Genetic Diversity Exploration (WIDDE), accessible at http://widde.toulouse.inra.fr/widde (Sempéré et al., 2015). Since the data were previously collected and publicly available, no specific ethics approval was required for this analysis. The dataset includes genetic information from 113 individuals of SSA cattle breeds, previously studied by Gautier et al. (2009), and was genotyped using the Illumina 50K SNP chip (Illumina Inc., San Diego, CA, United States). The chromosomal locations are based on the UMD 3.1 assembly of the bovine genome.

Details of the breeds studied are as follows:

• Kuri (KUR): 30 samples were collected from the Lake Chad islands. These cattle are traditionally managed under pastoral systems that capitalize on the extensive grazing available around Lake Chad (Moazami Goudarzi et al., 2001).
• N’dama (NDA): 17 samples were collected from the Samandeni Ranch in Burkina Faso. The NDA are known for their adaptability to agropastoral systems, which combine crop cultivation with cattle rearing. This breed is typically praised for its resilience to local diseases and environmental stressors. Extensive ancestral details are available in Souvenir Zafindrajaona et al. (1999).
• Zebu-Bororo (ZBO) and Zebu-Fulani (ZFU): 23 and 43 samples, respectively, sourced from Malanville in Benin. These breeds are extensively used for crossbreeding and adapted for draught, milk, and meat production. They are noted for their disease resistance, heat tolerance, and efficient resource utilization. Both breeds are managed under traditional pastoral systems, which involve extensive grazing and movement across different grazing areas, with production management practices described (Moazami Goudarzi et al., 2001) and further details about the breeds in other studies (Vanvanhossou et al., 2021a; Ouédraogo et al., 2021).

To provide a comparative perspective on the genetic diversity of SSA cattle, we also included individuals from subtropical and temperate breeds. The subtropical breeds studied were Gir (GIR), Sahiwal (SAH), and Tharparkar (THA), with 17, 24, and 12 samples, respectively. The temperate breeds included were Holstein (HOL) and Jersey (JER), with 64 and 28 samples, respectively. The genetic data for these breeds were also sourced from WIDDE and previous research (Matukumalli et al., 2009; Decker et al., 2014).

### 2.2 Runs of homozygosity and detection of genome-wide selection signatures

#### 2.2.1 Data preparation and quality control

Initially, pedigree (.ped) and map (.map) files were converted into binary format files (.bim, .bed, and.fam) using the --make-bed command in PLINK v1.9 software (Purcell et al., 2007). To ensure stringent quality control, we modified an R script written by Gorssen et al. (2021), which automated the application of specific quality control measures. Following their methodology, we retained only autosomal single nucleotide polymorphisms (SNPs) and set thresholds for individual call rates at a minimum of 90% (--mind 0.10) and SNP call rates at a minimum of 95% (--geno 0.05). We did not perform minor allele frequency pruning, Hardy-Weinberg equilibrium testing, or linkage disequilibrium pruning to maintain a comprehensive dataset conducive to ROH analysis (Meyermans et al., 2020). Before quality control, a common set of 51,998 SNPs covering the autosomes was extracted for each SSA cattle breed. These were then merged for cross-population analyses. After the application of quality control measures, the number of usable SNPs was reduced to 48,835. All individuals met the established quality criteria, resulting in no exclusions from the study.

#### 2.2.2 ROH analysis

After implementing initial quality control protocols, we conducted our ROH analysis using PLINK, guided by the methodologies suggested in Meyermans et al. (2020). To accurately identify ROH segments, we enforced strict criteria: no heterozygous SNPs were permitted within any ROH segment (--homozyg-window-het and--homozyg-het), and the allowance for a single missing SNP per window (--homozyg-window-missing) was made. The specific number of SNPs required per window (--homozyg-window-snp) and within each ROH segment (--homozyg-snp) was set based on the unique genetic traits of each breed, utilizing the L-parameter for guidance (Meyermans et al., 2020), with the settings adjusted to 61 for KUR, 66 for NDA, 65 for ZBO, and for 68 ZFU. Each window for ROH analysis was set at a minimum of 1,000 kb (--homozyg-kb), with a density requirement of one SNP every 150 kb (--homozyg-density). The maximum allowed gap between consecutive SNPs within an ROH was capped at 1,000 kb (--homozyg-gap), and window threshold of two outer SNPs ensuring continuity and integrity of the homozygous segments. Additionally, the proportion of each chromosome covered by ROH was calculated by dividing the mean ROH length per chromosome by its total length in Mb, providing normalized measurements of ROH coverage across the genome. Average SNP density was at least one SNP per 51 kb for all populations. This parameterization ensured that over 97% of the autosomal genome was covered, facilitating detection of ROH across the studied breeds. ROHs were categorized into four length classes to elucidate their potential genetic impacts: 1–4 Mb (short segments), 4–6 Mb (moderate segments), 6–8 Mb (long segments), and >8 Mb (very long segments).

We employed PLINK to calculate various genomic inbreeding coefficients to provide a multifaceted view of inbreeding. The genomic inbreeding coefficient ( $F_{ROH}$ ) was calculated using McQuillan et al. (2008) formula: $F_{ROH}=\sum L_{ROH}/L_{AUTO}$ , where $\sum L_{ROH}$ represents the total length of all ROH in the genome of an individual and $L_{AUTO}$ is the length of the autosomal genome covered by SNPs in the analysis. Additionally, we calculated three different estimates of the genomic inbreeding coefficient for each breed: $F_{GRM}$ based on the variance-standardized relationship - 1, $F_{\mathrm{HOM}}$ estimated from the excess of homozygotes, and $F_{UNI}$ derived from the correlation between uniting gametes (Yang et al., 2011). These coefficients $F_{GRM}$ , $F_{\mathrm{HOM}}$ , and $F_{UNI}$ , were computed using --ibc flag in PLINK. The genomic inbreeding coefficient for each breed was determined by averaging these coefficients across all individuals within the breed.

To evaluate differences in ROH, genomic inbreeding metrics, and genome coverage among cattle breeds, we conducted analysis of variance (ANOVA) followed by a Tukey-Kramer post-hoc analysis using the aov function for ANOVA and HSD.test functions from the R package agricolae v1.3-7 (Felipe, 2023). This approach allowed us to identify significant disparities at p<0.05 , accommodating comparisons across groups with unequal sample sizes. To further analyze the data, we conducted a correlation analysis to examine the relationships between the distributions of ROH and the various inbreeding metrics. We further explored the relationships between the distributions of ROH and various inbreeding metrics through correlation analysis. Employing the cor.test function in R, we calculated Pearson’s correlation coefficients and determined p -values at significance levels of 0.001, 0.01, and 0.05 to evaluate the statistical significance of the observed correlations. The correlation analysis was displayed using R package corrplot v0.92 (Wei and Viliam, 2021).

#### 2.2.3 Detection of ROH islands

Detection of ROH islands was performed using PLINK to quantify ROH incidence, defined as the percentage of animals within a population having a SNP within an ROH segment. The visualization of these incidences was done using Manhattan plots via the R package qqman v0.1.9 (Turner, 2018). ROH islands, indicative of positive selection, were defined based on SNPs exceeding a population-specific threshold. This threshold, derived from standard normal z-scores of ROH incidences, set a cutoff where the top 0.1% of SNPs with a p -value over 0.999 were considered significant (Purfield et al., 2012; Gorssen et al., 2021). A minimal incidence threshold of 20% was required for an ROH to qualify as an island as an additional restriction.

### 2.3 Detecting positive selection

To detect evidence of recent positive selection within SSA cattle populations, we employed the iHS using the R package rehh v3.2.2 (Gautier and Vitalis, 2012; Gautier et al., 2017). This statistical tool identifies alleles that have undergone selective sweeps by examining the extended haplotype homozygosity (EHH) around a core allele compared to its ancestral state. The iHS is particularly effective for pinpointing long haplotypes that appear more frequently than expected, suggesting recent positive selection.

We phased the genotype data for each chromosome of the *Bos taurus* populations using the SHAPEIT software (Delaneau et al., 2013), a crucial step for the subsequent iHS analysis (Voight et al., 2006). Following phasing, we determined the EHH for both the ancestral ( ${iHH}_{A}$ ) and derived alleles ( ${iHH}_{D}$ ) at each SNP with a minor allele frequency (MAF) of at least 5%. Then we calculated the un-standardized log-ratio ( uniHS ) for specific markers as:

$${uniHS}_{\left(s\right)}=\ln\left(\frac{{iHH}_{A\left(s\right)}}{{iHH}_{D\left(s\right)}}\right).$$

Following the methodology outlined by Voight et al. (2006), we standardized the uniHS scores to account for allele frequency variability using a frequency bin of 0.025:

$${iHS}_{\left(s\right)}=\frac{{uniHS}_{\left(s\right)}-mean\left({uniHSǀp}_{s}\right)}{sd\left({uniHSǀp}_{s}\right)}.$$

Here, $mean\left({uniHSǀp}_{s}\right)$ and $sd\left({uniHSǀp}_{s}\right)$ represent the mean and standard deviation of uniHS values for a bin of SNPs with similar derived allele frequencies at marker s . This standardization ensures that iHS values are approximately normally distributed, allowing valid comparisons across the genome.

We associated p -values with iHS scores to identify outliers and assess the significance of selection signals. The p -value for iHS ( piHS ) is defined as:

$$piHS=-\log_{10}\left(2ɸ\left(-\left|iHS\right|\right)\right),$$

where $ɸ\left(x\right)$ represents the Gaussian cumulative distribution function.

We then analyzed these iHS scores across the genome using sliding windows of 1 Mb, each overlapping the subsequent one by 100 kb. Each window required at least two extremal markers with scores exceeding a threshold. A threshold of 4, corresponding to a *p*-value of less than 0.0001, was set to identify significant selection signals, highlighting regions with significant genetic differentiation indicative of recent positive selection. Significant iHS scores are visualized using Manhattan plots generated by the same rehh package.

### 2.4 Gene annotation

Following the detection of ROH island regions and other genomic areas under positive selection identified through iHS analysis, we used the Ensembl Genes 112 database specific to *Bos taurus*. We input the genomic coordinates of these regions into the Ensembl genome browser (https://www.ensembl.org) to facilitate the extraction of detailed gene annotations. To automate the retrieval of gene information from Ensembl, we employed the R package biomaRt v2.60.0 (Durinck S et al., 2009). This enabled us to effectively query the Ensembl REST API, obtaining comprehensive gene annotations including gene symbols and descriptions of their products. This annotation process allows the identification of known and potentially novel genes that may contribute to adaptive traits in SSA cattle.

### 2.5 Genetic diversity estimation using principal component analysis (PCA)

We performed PCA to compare the genetic diversity of SSA cattle with subtropical and temperate breeds, facilitating a comprehensive evaluation of genetic adaptation across varied environmental conditions. PCA was conducted using PLINK software, employing a pruned dataset of SNPs to minimize the impact of linkage disequilibrium (Purcell et al., 2007). SNP pruning was performed with specific parameters: a window size of 50 SNPs, a step size of 10 SNPs per shift, and an $r^{2}$ threshold of 0.1. These settings ensure that only SNPs in approximate linkage equilibrium are included, providing clearer insights into the underlying genetic structure without the confounding effects of SNP correlation.

The output from PCA was visualized using the R package ggplot2 v3.5.0 (Wickham, 2016). This visualization process highlights the clustering and dispersion of genetic variability across the breeds studied, reflecting their evolutionary and geographical backgrounds. The generated plots facilitate the observation of distinct or overlapping genetic clusters among SSA, subtropical, and temperate cattle breeds.

## 3 Results

### 3.1 Genomic patterns of inbreeding and runs of homozygosity in SSA cattle

Table 1 summarizes the distribution of ROH and inbreeding coefficients across four SSA cattle breeds: KUR, NDA, ZBO, and ZFU. In total, 720 ROH segments were detected: 150 in KUR, 128 in NDA, 146 in ZBO, and 296 in ZFU (results not shown). The analysis revealed notable differences in the extent and distribution of ROH among the breeds. The mean number of ROH segments did not significantly vary across the breeds, with values ranging from 5.0 ± 0.71 in KUR to 7.53 ± 2.52 in NDA ( p=0.46 ). However, the distribution of these segments across different length categories showed statistical significance.

**TABLE 1:** Distribution of runs of homozygosity (ROH) and inbreeding coefficients among Sub-Saharan African cattle breeds.

Metric	Kuri	N’dama	Zebu-bororo	Zebu-fulani	*p*-value
ROH (Mean ± SE)	5 ± 0.711	7.529 ± 2.516	6.348 ± 1.017	6.884 ± 0.785	0.461
ROH 1–4 Mb (Mean ± SE)	2.133 ± 0.218	1.176 ± 0.346	2.565 ± 0.371	2.233 ± 0.315	0.084
ROH 4–6 Mb (Mean ± SE)	1.6 ± 0.286	1.235 ± 0.458	1.826 ± 0.293	2.442 ± 0.258	0.041*
ROH 6–8 Mb (Mean ± SE)	0.433 ± 0.114	0.882 ± 0.352	0.652 ± 0.173	0.907 ± 0.169	0.228
ROH >8 Mb (Mean ± SE)	0.833^b^ ± 0.413	4.235^a^ ± 1.542	1.304^b^ ± 0.687	1.302^b^ ± 0.376	0.01*
F_ROH_ (Mean ± SE)	0.013 ± 0.004	0.039 ± 0.015	0.018 ± 0.007	0.019 ± 0.004	0.086
F_ROH_ 1–4 Mb (Mean ± SE)	0.003 ± 0	0.002 ± 0.001	0.003 ± 0.001	0.003 ± 0	0.126
F_ROH_ 4–6 Mb (Mean ± SE)	0.003^ab^ ± 0.001	0.002^b^ ± 0.001	0.004^ab^ ± 0.001	0.005^a^ ± 0.001	0.024*
F_ROH_ 6–8 Mb (Mean ± SE)	0.001 ± 0	0.003 ± 0.001	0.002 ± 0	0.003 ± 0	0.219
F_ROH_ >8 Mb (Mean ± SE)	0.006^b^ ± 0.003	0.033^a^ ± 0.013	0.009^ab^ ± 0.006	0.008^b^ ± 0.004	0.023*
*F*_*GRM*_	−0.258^b^ ± 0.006	−0.485^a^ ± 0.024	−0.263^b^ ± 0.005	−0.256^b^ ± 0.011	<0.001*
*F*_*HOM*_	−0.009^b^ ± 0.006	−0.072^a^ ± 0.016	−0.002^b^ ± 0.007	−0.006^b^ ± 0.01	<0.001*
*F*_*UNI*_	−0.009^b^ ± 0.003	−0.072^a^ ± 0.009	−0.002^b^ ± 0.004	−0.006^b^ ± 0.002	<0.001*
ROH Genome coverage (Mb)	32.337 ± 9.903	96.682 ± 36.96	44.707 ± 16.077	46.824 ± 9.919	0.086

^a,b,c^ indicate statistical groupings; *F*_*ROH*_: ROH-based inbreeding coefficient; *F*_*GRM*_: genetic relationship matrix-based inbreeding coefficient; *F*_*HOM*_: inbreeding coefficient based on excess homozygosity; *F*_*UNI*_: inbreeding coefficient based on the correlation between uniting gametes; * = p<0.05.

ROH segments between 4–6 Mb demonstrated significant differences across breeds, with a range of 1.24 ± 0.46 in NDA and 2.44 ± 0.26 in ZFU ( p=0.041 ). However, despite this statistical significance, the post-hoc analysis did not reveal distinct differences between individual breeds. This indicates that while variability exists, it is not substantial enough to distinctly separate the breeds based on ROH segments within this size range. In contrast, longer ROH segments (>8 Mb) ranged from 0.83 ± 0.41 in KUR to 4.24 ± 1.54 in NDA, showing significant overall differences and clear distinctions between breeds in post-hoc tests. Notably, NDA exhibited a higher occurrence of these segments, which significantly differed from other breeds ( p=0.01 ).

In terms of inbreeding coefficients calculated from ROH ( $F_{ROH}$ ), these varied across breeds, with NDA recording the highest mean coefficient of 0.039 ± 0.015. Although these differences were not statistically significant ( p=0.084 ), the inbreeding coefficients related to specific ROH lengths (4–6 Mb and >8 Mb) highlighted significant differences. The $F_{ROH}$ for 4–6 Mb was highest in ZFU and significantly different from the lower values seen in NDA, while the >8 Mb category demonstrated a higher inbreeding impact in NDA relative to other breeds.

Additional inbreeding metrics such as the $F_{GRM}$ , $F_{\mathrm{HOM}}$ , and $F_{UNI}$ further elucidate the genetic structure within these cattle populations. Notably, NDA consistently displayed the lowest values across these metrics when compared to other breeds, with statistical significance ( p<0.001 ). The total genomic coverage by ROH varied across breeds with NDA showing the highest coverage at 96.68 ± 36.96 Mb, followed by ZFU (46.82 ± 9.912 Mb), ZBO (44.707 ± 16.077 Mb), and KUR (32.337 ± 9.903 Mb), although these differences were not statistically significant ( p=0.086 ).

**FIGURE 1:**
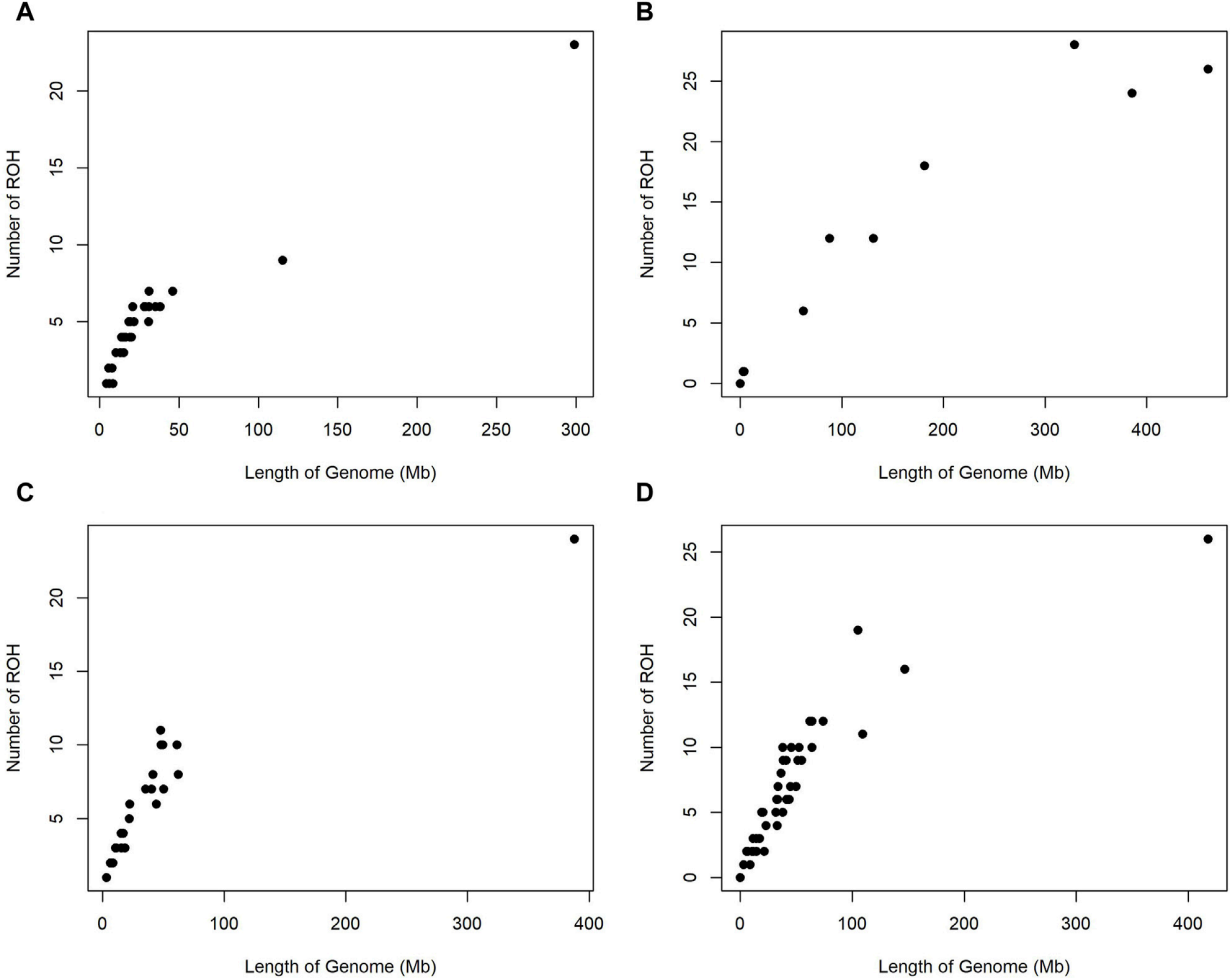
Scatter plots depicting the relationship between the number of runs of homozygosity (ROH) per individual and the cumulative genomic length covered by ROH across four Sub-Saharan African cattle breeds: Kuri **(A)**, N’dama **(B)**, Zebu-Bororo **(C)**, and Zebu-Fulani **(D)**.

Figure 1 illustrates the individual distribution of ROH in relation to the genomic length covered by these segments for four cattle breeds. For the KUR breed (panel A), a dense clustering of points within a narrow genomic length suggests a uniform extent of ROH across individuals. The NDA breed (panel B) shows a widespread coverage of ROH, indicating a heterogeneous pattern of genomic regions affected by inbreeding. The ZBO (panel C) and ZFU (panel D) breeds demonstrate a more dispersed set of points, indicating a broader range of inbreeding influences, with the ZFU exhibiting a larger number of ROH segments extending over greater genomic lengths.

### 3.2 Correlation analysis of inbreeding metrics within SSA cattle populations

**FIGURE 2:**
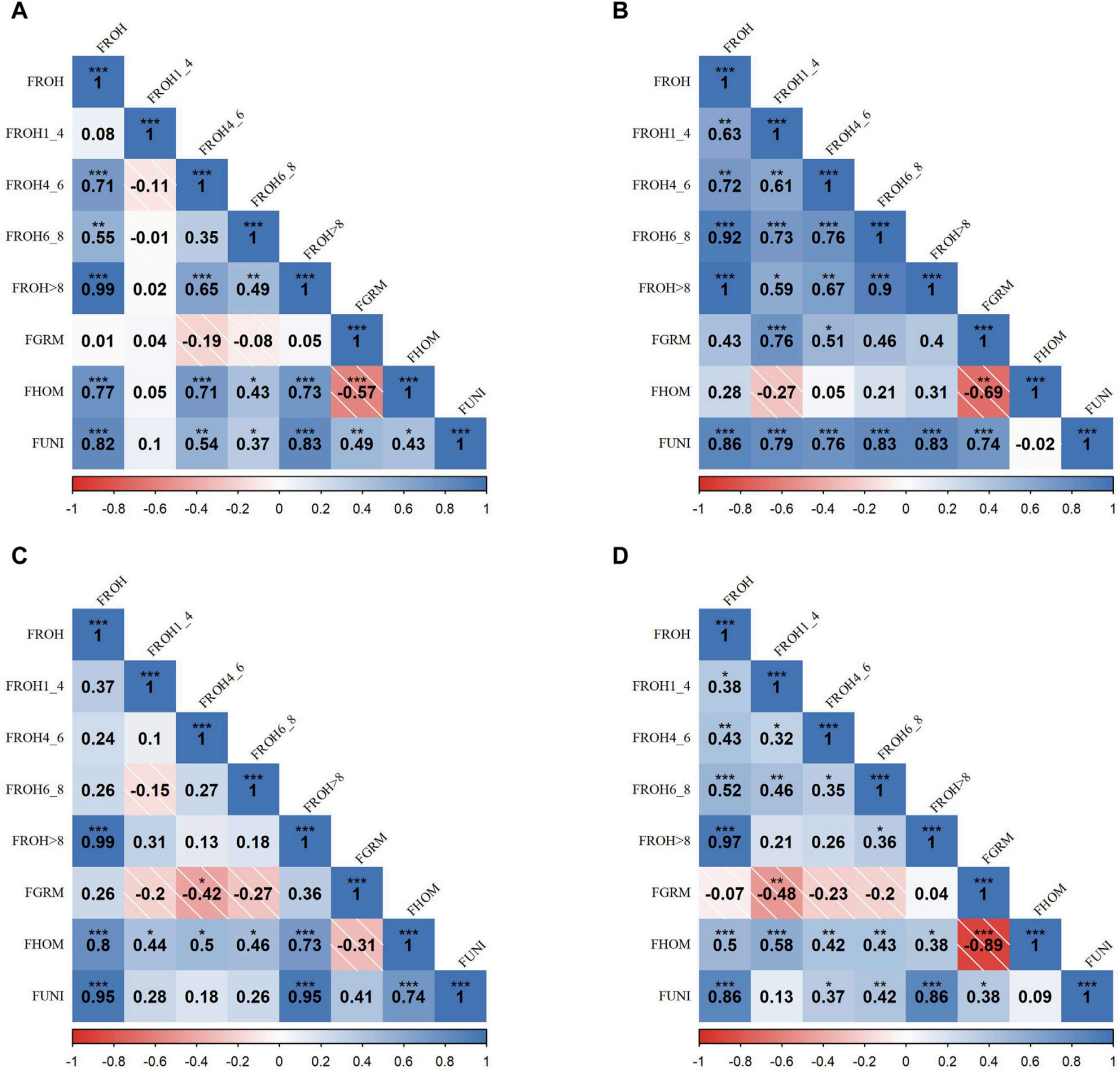
Pearson correlation matrices for inbreeding metrics and ROH categories across four Sub-Saharan African cattle breeds: Kuri **(A)**, N’dama **(B)**, Zebu-Bororo **(C)**, and Zebu-Fulani **(D)**. The matrices display correlations between different inbreeding metrics, including ROH-based inbreeding coefficients (*F*_*ROH*_) for segment lengths (all, 1–4 Mb, 4–6 Mb, 6–8 Mb, >8 Mb), genetic relationship matrix-based inbreeding coefficient (*F*_*GRM*_), inbreeding coefficient based on excess homozygosity (*F*_*HOM*_), and inbreeding coefficient from correlation between uniting gametes (*F*_*UNI*_). Positive correlations are shown in blue and negative in red, with statistical significance as *=0.05 , **=0.01 , ***=0.001 , indicating increasing significance levels.

Figure 2 presents the correlation matrices that detail the relationships between various inbreeding metrics across four SSA cattle breeds. In the KUR breed (A), $F_{ROH}$ showed a strong correlation with both $F_{\mathrm{HOM}}$ ( r=0.77 ; p<0.001 ) and $F_{UNI}$ ( r=0.82 ; p<0.001 ), indicating a significant relationship between these metrics of inbreeding. However, $F_{GRM}$ was virtually uncorrelated with $F_{ROH}$ ( r=0.01 ), suggesting it captures different genetic structure aspects. In NDA breed (B), correlations within $F_{ROH}$ categories were notably high (ranging from 0.59 to 1, p<0.05 ), especially for longer segments, with strong associations with $F_{UNI}$ ( r=>0.83 ; p<0.001 ). A significant negative correlation between $F_{\mathrm{HOM}}$ and $F_{GRM}$ ( r=−0.69 ; p<0.01 ) was observed, indicating divergent influences on these metrics.

In the ZBO breed (C), there were substantial correlations between $F_{ROH}$ and segments longer than 8 Mb ( r=0.99 ; p<0.001 ), $F_{\mathrm{HOM}}$ ( r=0.80 ; p<0.001 ), and $F_{UNI}$ ( r=0.95 ; p<0.001 ). Limited negative correlations between $F_{GRM}$ and other metrics highlight distinct patterns of genetic structure relative to $F_{ROH}$ and $F_{\mathrm{HOM}}$ . For ZFU breed (D), notable correlation existed between $F_{ROH}$ , especially for longer segments ( r=>0.5 ; p<0.001 ), and $F_{UNI}$ ( r=0.86 ; p<0.001 ). $F_{\mathrm{HOM}}$ showed a significant negative correlation with $F_{GRM}$ ( r=−0.89 ; p<0.001 ), highlighting discrepancies in genetic similarity interpretations within the breed.

Overall, $F_{ROH}$ correlates positively with $F_{\mathrm{HOM}}$ and $F_{UNI}$ across all breeds, indicating that increased homozygosity aligns with higher inbreeding coefficients. Conversely, the correlation between $F_{ROH}$ and $F_{GRM}$ is generally weak or negative, suggesting $F_{GRM}$ captures broader genetic variance not solely explained by homozygosity levels. These contrasts, particularly the negative correlations between $F_{GRM}$ and $F_{\mathrm{HOM}}$ , indicate that these metrics reflect distinct aspects of genetic structure.

### 3.3 Runs of homozygosity distribution and chromosome coverage

**FIGURE 3:**
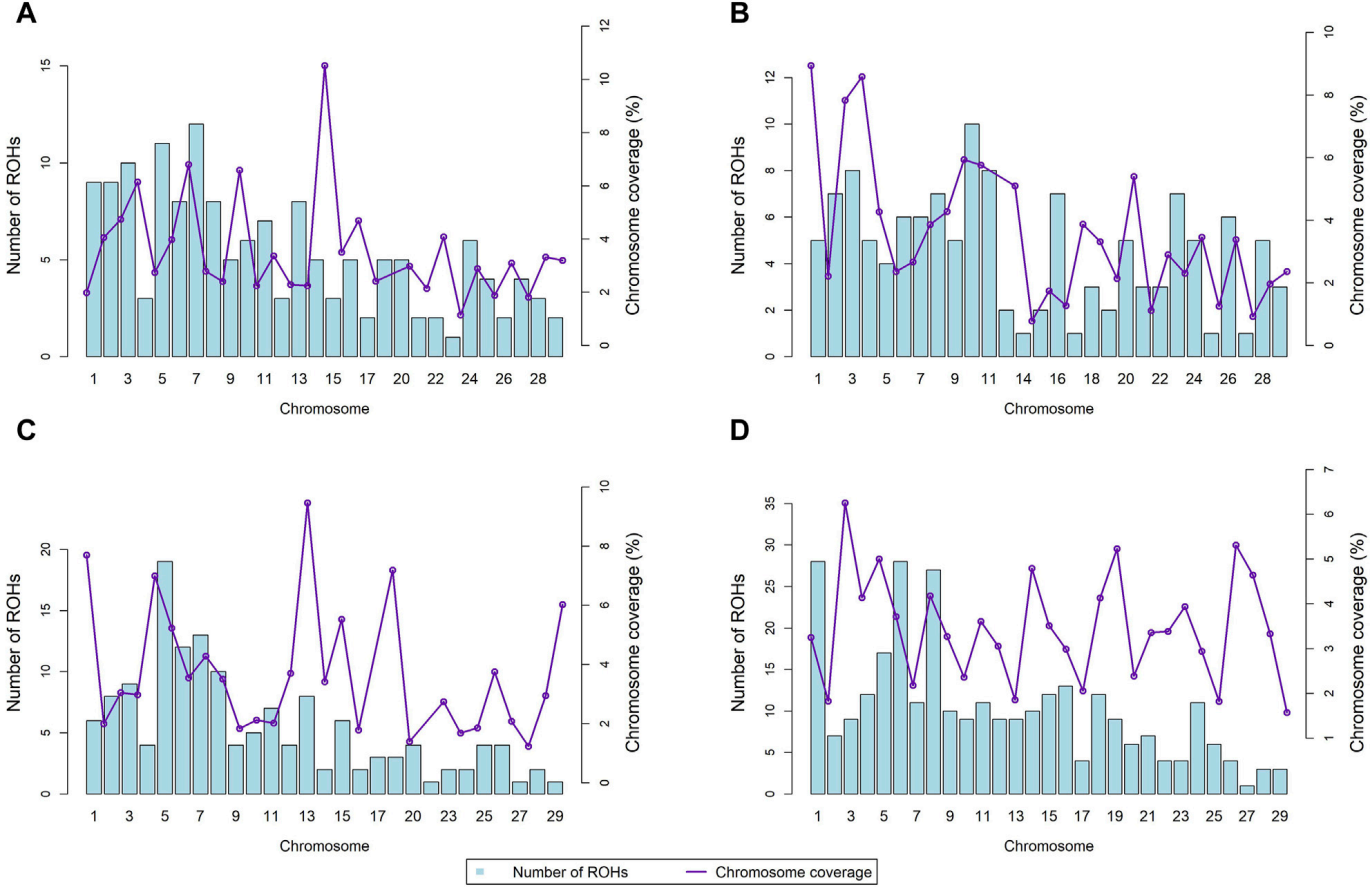
Chromosomal distribution and coverage of runs of homozygosity (ROH) across four Sub-Saharan African cattle breeds: Kuri **(A)**, N’dama **(B)**, Zebu-Bororo **(C)**, and Zebu-Fulani **(D)**.

Figure 3 depicts the chromosomal distribution and coverage of ROH for four SSA cattle breeds, indicated by bar graphs and overlaid line graphs, respectively. For the KUR breed (Panel A), ROHs were most concentrated on chromosomes 7, 5, and 3, with counts of 12, 11, and 10, respectively. Chromosome 15 showed the highest genomic coverage at approximately 10.52%, whereas the lowest coverage was observed on chromosome 23 at about 1.14%. In the NDA breed (Panel B), chromosome 10 displayed the highest ROH count with 10 segments, closely followed by chromosomes 11 and 3, each also registering 8 segments. The greatest coverage was on chromosome 1 at 8.94%, with the smallest coverage on chromosome 14, recording only 0.78%. The ZBO breed (Panel C) exhibited a broad spread of ROHs, notably on chromosomes 5 and 7, with 19 and 13 segments respectively. The highest coverage was on chromosome 5 at approximately 6.99%, and the lowest was on chromosome 27 with 1.23%. For the ZFU breed (Panel D), there was a significant accumulation of ROHs on chromosomes 1, 6, and 8, each showing at least 27 segments. Chromosome 3 had notable coverage at about 6.25%, while chromosome 29 showed the least coverage at roughly 1.57%.

### 3.4 ROH island and integrated haplotype scores revealed potential candidate genes related to breed characteristics in SSA cattle

**FIGURE 4:**
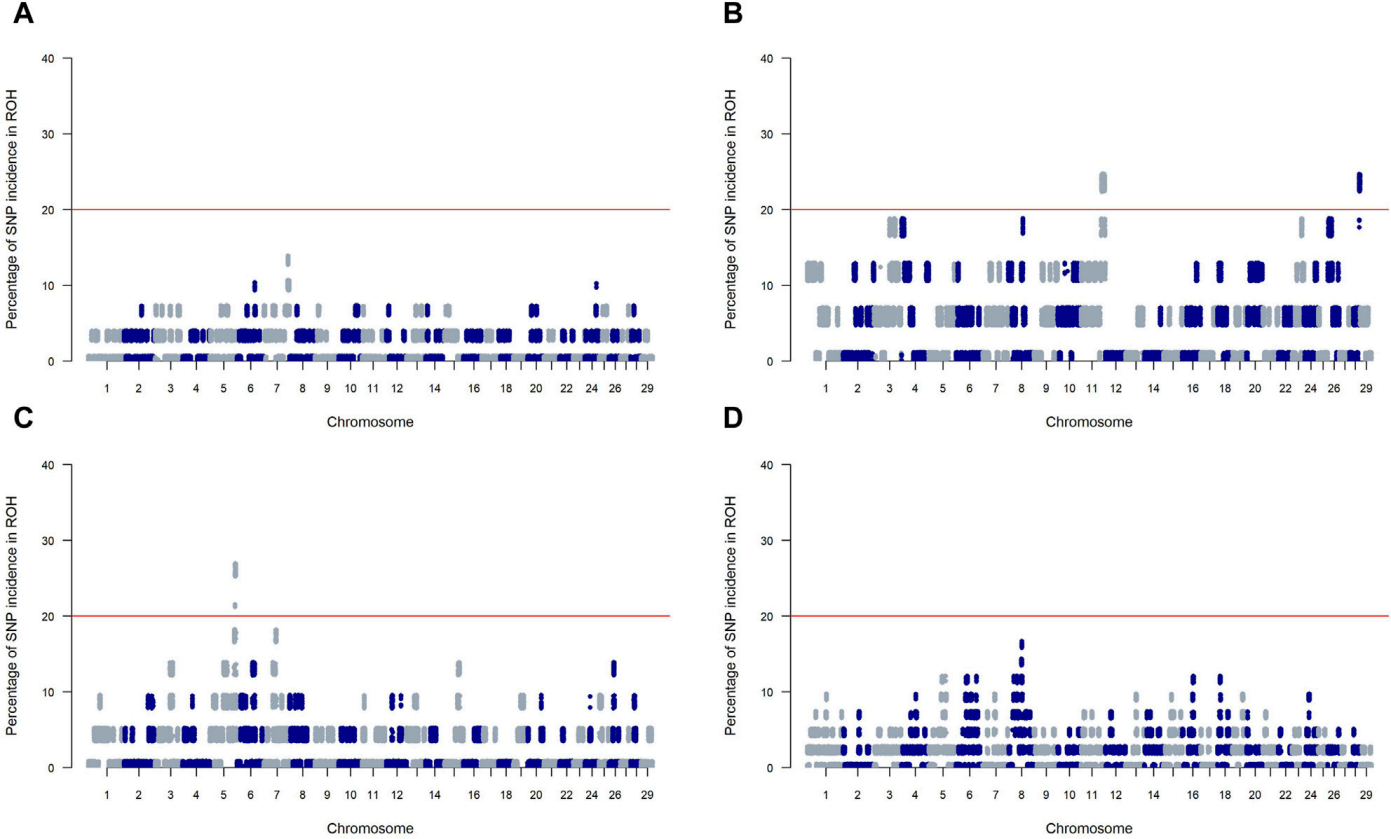
Manhattan plots of SNP incidence within runs of homozygosity (ROH) across four Sub-Saharan African cattle breeds: Kuri **(A)**, N’dama **(B)**, Zebu-Bororo **(C)**, and Zebu-Fulani **(D)**. A red line marks the 20% threshold for identifying significant ROH islands.

Figure 4 presents Manhattan plots for SNP incidence within ROH across four SSA cattle breeds. The red horizontal line at the 20% mark on each plot is a threshold for distinguishing significant ROH islands. KUR (Panels A) and ZFU (Panel D) show few incidences below the threshold, indicating a scattered presence of significant ROH islands within these breeds. Conversely, NDA (Panel B) and ZBO (Panel C) display multiple chromosomal locations where the incidence of SNPs surpasses the 20% threshold, which suggests a greater presence of significant ROH islands.

Our genomic analysis detected 16 and 5 candidate regions on chromosomes 11 and 28, respectively, in the NDA breed, and 5 regions on chromosome 5 of the ZBO breed. From these regions, a total of 308 genes symbols were identified, as detailed in Supplementary Table S1. Among these, 14 are represented solely by marker names and lack functional annotations. Notable among the functionally annotated genes are *RSAD2*, *CMPK2*, *SOX11*, *GGTA1*, *OR1J1*, *DENND1A*, and *NOTCH1* on chromosome 11; and *ARHGAP22* and *CXCL12* on chromosome 28 for NDA. In the ZBO breed, significant genes on chromosome 5 include *SLC25A17*, and *EFCAB6*.

**FIGURE 5:**
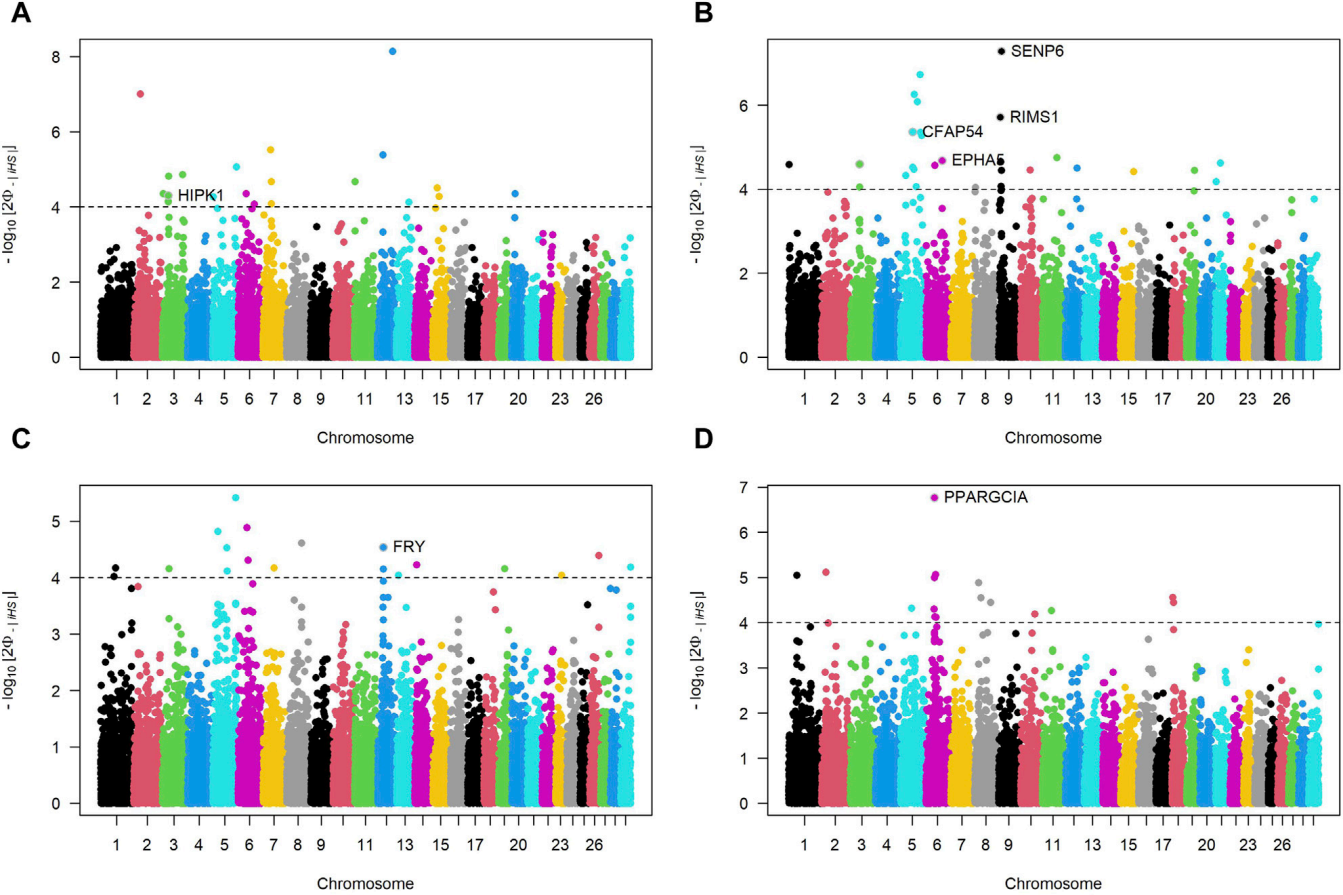
Manhattan plots of Integrated Haplotype Scores (iHS) across autosomal chromosomes for four Sub-Saharan African cattle breeds: Kuri **(A)**, N’dama **(B)**, Zebu-Bororo **(C)**, and Zebu-Fulani **(D)**. The plots illustrate -log10 P -values for iHS, identifying regions under positive selection. Horizontal dashed lines indicate the suggestive significance thresholds for selection signals.

Figure 5 depicts Manhattan plots of iHS for cattle breeds, where each dot represents a SNP. The dashed horizontal lines denote suggestive thresholds for positive selection signals, with data points above these lines indicating regions potentially under selection.

For the KUR breed (Panel A), a notable peak is present on chromosome 3, where the gene *HIPK1* is identified as a candidate for positive selection. The NDA breed (Panel B) exhibits multiple regions with significant iHS signals on several chromosomes, including strong peaks associated with genes like *EPHA5, CFAP54, SENP6* and *RIMS1*. The ZBO breed (Panel C) shows a pronounced selection signal on chromosome 12, with the gene FRY highlighted. The ZFU breed (Panel D) has a distinct peak on chromosome 6, where *PPARGC1A* is identified among the candidate genes. Supplementary Table S2 complements these iHS findings by providing a detailed summary of the candidate genomic regions, including the bovine chromosome number (BTA), the specific locations of these regions, gene symbols, and products. For instance, it elucidates that the NDA breed has putative sweeps on chromosomes 5, 6, and 9, including *ELK3, HTR1B,* and *MEI4*, suggesting these areas have undergone selection sweeps. Similarly, for the ZBO, the table highlights a large genomic region on chromosome 12 with 2939 exons and several candidate genes, such as *BRCA2* and *ALOX5AP*, indicating a substantial genomic section subject to selection.

### 3.5 Genetic diversity using principal component matrix

**FIGURE 6:**
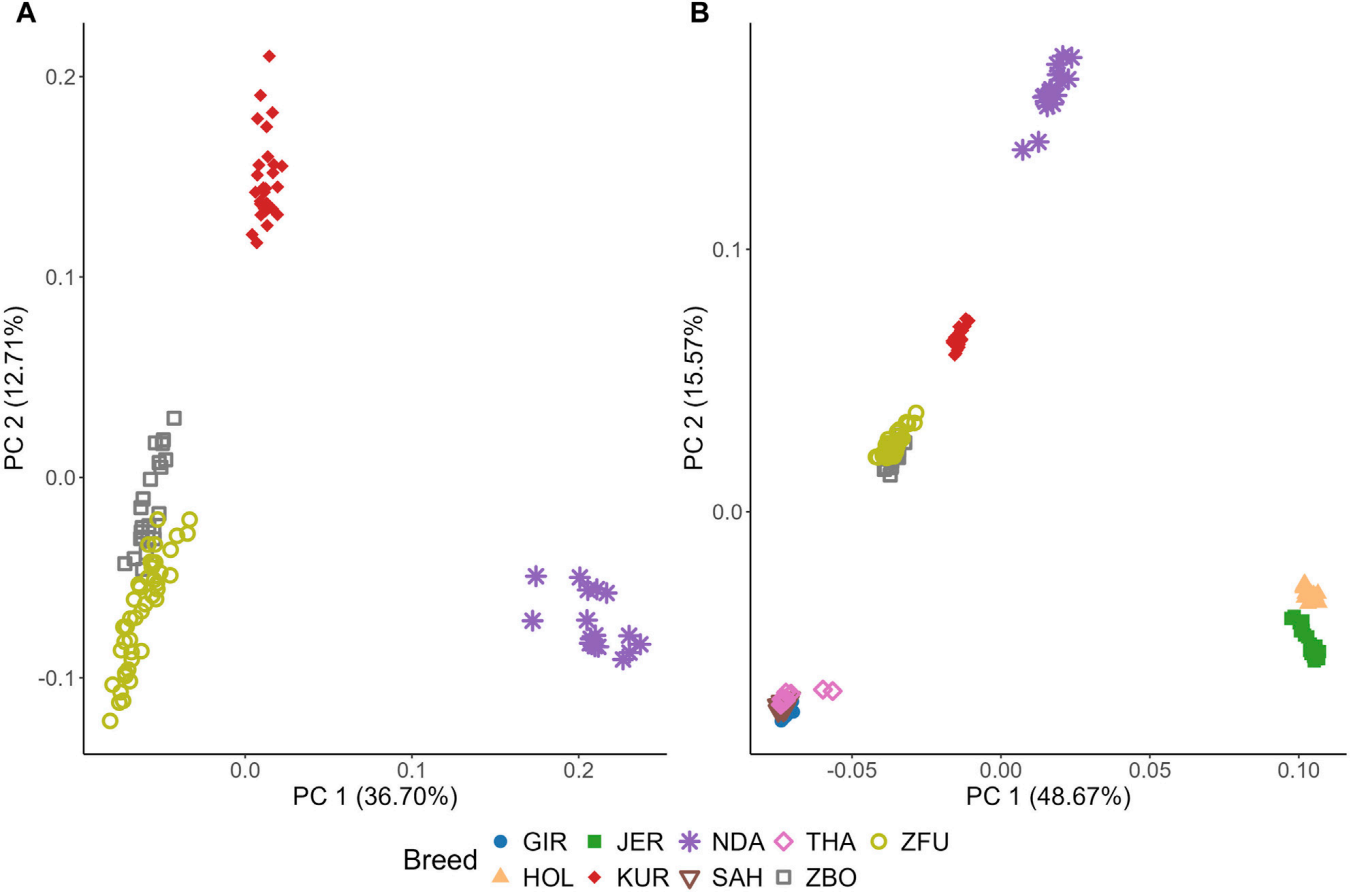
Principal component (PC) analysis (PCA) of genetic diversity in cattle breeds: Sub-Saharan African breeds **(A)** and breeds from diverse climatic regions **(B)**. KUR: Kuri, NDA: N’dama, ZBO: Zebu-Bororo, ZFU: Zebu-Fulani, GIR: Gir, SAH: Sahiwal, THA: Tharparkar, HOL: Holstein, JER: Jersey.

Figure 6 illustrates the genetic diversity among cattle breeds through PCA. In Figure 6A, focusing on SSA cattle, PC1 and PC2 captured 36.70% and 12.71% of the genetic variance, respectively, as detailed in Supplementary Figure S1. KUR forms a distinct and tight cluster, indicative of high genetic homogeneity. NDA also forms a clear cluster but shows more dispersion than KUR, suggesting higher genetic variability within this breed. Notably, ZBO and ZFU exhibit significant overlap, indicating their close genetic relationship and shared genetic background.

Contrasting with Figures 6A, B includes breeds from subtropical and temperate regions, showing that PC1 and PC2 explain a larger proportion of the variance, at 48.67% and 15.57%, respectively. Among the subtropical breeds, GIR, SAH, and THA form a distinct cluster with significant overlap, highlighting a close genetic relationship. Meanwhile, the temperate breeds, HOL and JER, are closely positioned yet form separate clusters with no overlap, underscoring their unique genetic characteristics. HOL is particularly noted for its tight clustering, suggesting high genetic homogeneity.

## 4 Discussions

This study used comprehensive genomic tools to explore genetic diversity and adaptive mechanisms among SSA cattle, commonly found within pastoral and agropastoral systems. These environments are integral to the region’s livelihoods and cultural heritage, imposing unique environmental and selective pressures that shape the cattle’s genetic structure. We have revealed the complex interplay between inbreeding effects and selective adaptations through genomic analyses, including ROH and heterozygosity assessments. Our methodology underscores the resilience and adaptability of these indigenous breeds, filling significant knowledge gaps regarding how genetic diversity supports their survival under varied and challenging conditions. The insights gained are pivotal for informing sustainable livestock management and genetic conservation strategies, enhancing SSA food security and economic stability.

### 4.1 Genomic patterns of inbreeding and runs of homozygosity in SSA cattle

Our analysis of ROH and inbreeding coefficients across four SSA cattle breeds offers valuable insights into their genetic structure influenced by both historical and contemporary breeding practices (Table 1; Figure 1). The KUR breed displayed a significant prevalence of shorter ROH segments, generally under 4 Mb, suggesting ancient inbreeding or shared ancestry (Purfield et al., 2012). These genetic signatures indicate a long-standing genetic stability likely fostered by extensive pastoral systems. Such systems have historically promoted genetic diversity through natural migration and minimal human-directed breeding, suggesting a breed well-adapted through natural selection to its environment (Orenge et al., 2012; Taye et al., 2017; Kim et al., 2020; Xia et al., 2023). This adaptation process reflects the breed’s well-suited nature to its environment, aligning with the findings from Ouédraogo et al. (2021), which describe moderate levels of genomic inbreeding within local cattle populations, suggesting a balanced management of inbreeding akin to some well-managed European breeds.

In contrast, the NDA breed shows a higher occurrence of longer ROH segments, particularly those exceeding 8 Mb. These variations in ROH segments indicate recent inbreeding (Purfield et al., 2012; Sumreddee et al., 2021). Despite these indicators of recent inbreeding or intense selection for specific traits, the overall genetic diversity of the breed remains high, as indicated by non-significant overall inbreeding coefficients ( $F_{ROH}$ ) and supported by negative values in other inbreeding metrics ( $F_{GRM}$ , $F_{\mathrm{HOM}}$ , $F_{UNI}$ ). This pattern underscores the breed’s resilience and adaptability, potentially enhanced by agropastoral systems that encourage genetic variation through less stringent selection pressures and diverse mating practices (Souvenir Zafindrajaona et al., 1999; Kim J. et al., 2017). This aligns with the findings from Gautier et al. (2009) that document the genetic differentiation among cattle influenced by different breeding strategies.

The ZBO and ZFU breeds showcase a mixture of genetic influences, with a prominent distribution of medium-length ROH segments (4–6 Mb). These segments, statistically significant but not distinct enough to separate the breeds *post hoc*, reflect past inbreeding or shared ancestry. This genetic pattern is likely influenced by traditional pastoral systems where extensive grazing and movement facilitate genetic exchange, supporting the development of traits like drought resistance and enhanced milk production commonly selected in these breeds.

It is imperative to blend traditional knowledge with modern genomic tools to manage genetic diversity effectively, which includes establishing breed registries, employing rotational mating systems, and introducing new genetic material to preserve breed characteristics and enhance diversity (Eusebi et al., 2020; Oldenbroek, 2021). Conservation programs, such as those implemented for Ankole cattle in Uganda, demonstrate the effectiveness of integrating scientific plans with community participation to boost cattle productivity and genetic health (Nabasirye, 2012).

### 4.2 Correlation analysis of genomic inbreeding metrics within SSA cattle populations

Our analysis across SSA cattle breeds detailed the interactions between various genomic inbreeding metrics, providing essential insights into the genetic structure of these populations (Figure 2). This study highlights the critical role of different inbreeding coefficients in assessing the genetic makeup and historical breeding practices of cattle.

Strong correlations observed between $F_{ROH}$ , $F_{\mathrm{HOM}}$ , and $F_{UNI}$ across all breeds indicate their effectiveness in capturing homozygosity, which correlates with inbreeding levels that have accumulated over generations. Zhang et al. (2015) differentiate the implications of short and long ROH, noting that shorter ROH segments generally indicate ancient inbreeding, while longer segments reflect more recent inbreeding events. This understanding is crucial as it elucidates how different lengths of ROH can provide insights into the temporal dynamics of inbreeding within these populations.

In contrast, the consistently low correlation between $F_{ROH}$ and $F_{GRM}$ across breeds is particularly interesting. $F_{GRM}$ ’s sensitivity to allele frequencies allows it to capture a broader spectrum of genetic variation, which is not solely tied to homozygosity (Zhang et al., 2015). This metric’s ability to highlight genetic diversity beyond inbreeding is valuable for identifying genetic resilience and potential areas for genetic conservation or enhancement within breeding programs.

The negative correlation between $F_{\mathrm{HOM}}$ and $F_{GRM}$ , especially noted in breeds like the NDA, underscores the contrasting genetic insights these metrics provide. While $F_{\mathrm{HOM}}$ tends to indicate increased homozygosity, $F_{GRM}$ identifies regions of genetic heterogeneity, suggesting that these breeds retain significant genetic diversity despite historical inbreeding (Zhang et al., 2015). This divergence is crucial for maintaining breed health and adaptability, as it suggests that pockets of genetic diversity exist which can be critical for long-term breed sustainability.

These correlation trends offer valuable insights into how different inbreeding metrics can be interpreted in the context of breed management. They highlight the importance of using a combination of these metrics to gain a comprehensive understanding of both inbreeding levels and the broader genetic health of the breeds, which is crucial for making informed decisions in breeding programs aimed at maintaining genetic diversity and vigor.

### 4.3 Runs of homozygosity distribution and chromosome coverage

The distribution of ROH across SSA cattle breeds underscores historical selection pressures and provides crucial insights into contemporary genetic management and evolutionary strategies shaping these populations. The variance in ROH coverage among breeds such as KUR and NDA highlights regions under intense selection, likely reflecting adaptations to environmental pressures like disease resistance and drought tolerance (Dixit et al., 2020). Conversely, breeds such as ZBO and ZFU, exhibiting extensive ROH across multiple chromosomes, might represent strategies to enhance multi-trait resilience, which is beneficial for breeds in varied agricultural roles (Vanvanhossou et al., 2021b). This widespread selection could have evolved through breeding practices focused on developing robust cattle capable of thriving under diverse environmental conditions.

Areas with lower ROH indicate preserved genetic diversity, essential for a breed’s adaptability to future challenges like climate change or emerging diseases (Zhang et al., 2015; Illa et al., 2024). On the other hand, high-density areas of ROH might signal genetic bottlenecks or extensive inbreeding, potentially predisposing breeds to genetic disorders or limiting their adaptability to new stressors.

The detailed understanding of these chromosomal locations with high ROH can guide genomic selection efforts, allowing breeders to enhance traits linked to these areas while introducing genetic variability to mitigate inbreeding risks (Sonesson et al., 2012; Dixit et al., 2020; Meuwissen et al., 2020; Illa et al., 2024). For example, targeted crossbreeding programs could introduce fresh genetic material into high ROH areas, enhancing genetic health and trait diversity.

The patterns observed in ROH distribution provide a foundation for genetic studies in similar contexts and emphasize the importance of conservation strategies that balance trait enhancement with genetic diversity preservation. Initiatives such as gene banks and other genetic conservation measures are vital for maintaining the genetic heritage of these breeds, ensuring their long-term sustainability (Mapiye et al., 2019; Tenzin et al., 2023).

### 4.4 ROH island and integrated haplotype scores revealed potential candidate genes related to breed characteristics in SSA cattle

The integration of ROH and iHS analyses elucidates the complex genetic landscape of SSA cattle, emphasizing the influence of historical and contemporary selection pressures in defining breed-specific traits. This genetic overview, illustrated in Figures 4, 5 and elaborated in Supplementary Tables S1, S2, traces the evolutionary paths shaped by varied environmental and human-related factors.

For the NDA and ZBO breeds, significant ROH islands highlight regions of intense historical selection likely spurred by environmental challenges, as documented by Purfield et al. (2012). In contrast, the KUR and ZFU breeds exhibit fewer ROH islands, suggesting a history of milder selection pressures. This could indicate a broader genetic diversity within these breeds, possibly due to extensive mating pools common in pastoral and agropastoral systems (Moazami Goudarzi et al., 2001; Gautier et al., 2009; Gorssen et al., 2021). Such diversity might provide these breeds with a greater capacity to adapt to fluctuating environmental conditions, essential for managing future climatic changes.

In the NDA breed, genes located within ROH islands like *RSAD2* and *CMPK2* could indicate a genetic predisposition towards enhanced disease resistance, crucial for survival in areas burdened by endemic diseases. *RSAD2* plays a role in antiviral defense and immune responses, especially during pregnancy (Rocha et al., 2023). *CMPK2* has been associated with immune function and disease resistance, such as its involvement in bovine digital dermatitis and antiviral responses (Lai et al., 2021; Oelschlaegel et al., 2022). Previous studies in West African cattle have identified adaptive selection footprints linked to immune responses in West African taurine breeds, paralleling our discovery of *RSAD2* and *CMPK2* genes in NDA cattle, which suggest enhanced disease resistance (Gautier et al., 2009; Tijjani et al., 2019).

The *GGTA1* and *OR1J1* genes have potential roles in NDA cattle. *GGTA1*, encoding alpha-1,3-galactosyltransferase, is significant in immune modulation and various biological processes, particularly in xenotransplantation (Day and Rocha, 2008). Its role in NDA cattle may involve enhancing immune responses, though this remains speculative. *OR1J1*, a key gene in the olfactory system, potentially enhances foraging efficiency. This gene is regulated by the *MOR4* motif and shows expression differences among bulls, steers, and heifers (Lee et al., 2013; Kubo et al., 2016; Samuel and Dinka, 2020). It is crucial for feed appetence (Roura et al., 2008), suggesting an adaptive advantage in extensive pastoral landscapes.

Furthermore, the NDA breed shows potential genetic adaptations for physical resilience and metabolic efficiency, vital for breeds used in labor-intensive pastoral tasks. The *ARHGAP22* gene plays a crucial role in regulating actin dynamics, affecting synaptic plasticity and cognitive function in mice (Longatti et al., 2021). While its specific role in cattle is not well-documented, it could enhance cellular adaptability to physical stresses in NDA cattle. The inclusion of genes such as *NOTCH1*, with its role in developmental processes, might suggest its contribution to cellular differentiation and organ development. *NOTCH1* regulates cell proliferation and maintains the expression of key genes in early embryonic development (Li et al., 2021). In mice, disruption of *NOTCH1* leads to widespread cell death in embryos, indicating its essential role in post implantation development (Swiatek et al., 1994). These findings suggest that *NOTCH1* likely plays a similar role in cattle, supporting cellular differentiation and organ development, which are crucial for the breed’s overall health and productivity.

For ZBO cattle, genes like *SLC25A17* has been associated with higher milk yields in Chinese Holstein cattle (Lv et al., 2011), suggesting a role in metabolic efficiency. *EFCAB6* plays a crucial role in lipid metabolism and adipocyte proliferation (Chen et al., 2017; Junjvlieke et al., 2019). These genes likely contribute to breed’s survival in environments with limited resources.

The Manhattan plots of iHS provide insights into the ongoing selection pressures and identify genes critical for adaptive traits across SSA cattle breeds, enhancing our understanding of their genetic basis for adaptability and resilience.

In KUR cattle, the *HIPK1* gene emerges as a key factor in stress response adaptability, potentially aiding in coping with environmental extremes such as drought and high temperatures around Lake Chad. *HIPK1* plays a crucial role in cell growth and stress responses, including activating p53 to manage cellular stress (Isono et al., 2006; Rey et al., 2013). While the link between *HIPK1* and environmental stressors has not been directly studied, its role in cellular stress responses suggests it may help KUR cattle cope with harsh enviroments, which is crucial for breeds exposed to variable and harsh climatic conditions.

For NDA cattle, adapted to the mixed agropastoral systems of Burkina Faso, genes like *EPHA5* and *RIMS1*, linked to neural development and synaptic functions. *EPHA5* is involved in neural connectivity and immune response modulation (Fu et al., 2010; Staquicini et al., 2015; Huang et al., 2021). *RIMS1*, is crucial for neurotransmitter release and synaptic plasticity, impacting cognitive abilities (Sisodiya et al., 2007; Kaeser et al., 2008). Given their roles in neural and synaptic functions, it is hypothesized that *EPHA5* and *RIMS1* could influence complex behaviors in cattle, such as navigational abilities and social hierarchy management. These behaviors require complex neural processing, which these genes are known to support. Additionally, genes such as *SENP6* and *MYO6*, associated with apoptosis and cellular transport, may contribute to the breed’s resilience against diseases like trypanosomiasis, common in their geographic area. *MYO6* plays a crucial role in muscle development in other species (Tan et al., 2024), suggesting a similar function in cattle, potentially impacting overall health and disease resilience. *SENP6* is involved in genome stability, inflammation regulation, and apoptosis, essential for managing cellular stress during infections (Wagner et al., 2019; Mao et al., 2022). These roles in cellular processes suggest that *SENP6* and *MYO6* might enhance the breed’s resilience to diseases.

For ZBO cattle, genes like *FRY* and *ZAR1L*, identified through iHS analysis, may support traits linked to reproductive efficiency and cellular organization, essential under the extensive pastoral systems prevalent in their native Benin. *FRY* plays a crucial role in kidney development and function in mice (Byun et al., 2018), with potential involvement in fertility and milk production traits in cattle (Cai et al., 2019). *ZAR1L*, a maternal factor, is expressed during various stages of embryonic development, indicating its role in these processes (Brevini et al., 2004). The related *ZAR1*-like gene supports its role in oocyte development (Sangiorgio et al., 2008). These genes likely contribute to reproductive efficiency and cellular organization in ZBO cattle.

For ZFU cattle, the *PPARGC1A* gene is highlighted for its association with milk fat synthesis and overall milk composition traits, including fat and protein yield, critical for breeds extensively used for dairy production under challenging environmental conditions (Weikard et al., 2005; Pasandideh et al., 2015).

### 4.5 Genetic diversity using principal component matrix

The PCA analysis conducted in this study provides a comprehensive overview of the genetic diversity and structural relationships among various cattle breeds, with a focus on SSA breeds alongside subtropical and temperate counterparts, as depicted in Figure 6. This analytical approach reveals distinct genetic clusters, effectively illustrating the variations across different geographical and breeding contexts.

In the SSA group, the KUR breed displays a high degree of genetic homogeneity, which can be attributed to the pastoral systems prevalent around Lake Chad. These systems support natural migration and exert minimal selective pressures on breeding, resulting in a tightly clustered genetic profile. Such uniformity could confer adaptive advantages, allowing for stable existence in relatively undisturbed natural environments (Vanvanhossou et al., 2021b). On the other hand, the NDA breed demonstrates a more dispersed genetic pattern on the PCA, suggesting greater genetic variability. This is likely due to the breed’s integration into agropastoral systems that promote genetic diversity through random mating, thereby bolstering resilience against environmental and climatic challenges.

The genetic overlays of the ZBO and ZFU breeds indicate a closely knit genetic relationship, likely stemming from historical interbreeding and similar pastoral management practices. Adapted to various agricultural roles, these breeds exhibit traits that are advantageous in diverse and demanding environments, indicative of a strategic breeding approach to sustain a resilient genetic foundation (Vanvanhossou et al., 2021b).

Contrastingly, subtropical breeds such as the GIR, SAH, and THA exhibit tight clustering, reflecting their shared adaptations for heat tolerance and disease resistance. These traits are essential for thriving in subtropical climates and are a result of selective breeding processes focused on enhancing survival in hot environments (Dixit et al., 2020; Strucken et al., 2021). In a stark difference, temperate breeds like HOL and JER are distinctly separated on the PCA, showcasing the influence of intensive selective breeding aimed at optimizing traits like milk production. These breeds, developed under controlled breeding programs, highlight the divergent genetic paths taken to maximize agricultural productivity in temperate zones (Dixit et al., 2020; Xia et al., 2023).

### 4.6 General discussion

The genomic analysis delineated in this study highlight the crucial role of genetic diversity and inbreeding in shaping the adaptability and resilience of SSA cattle. We acknowledge certain methodological limitations, including reliance on SNP chips, which might not capture the entire spectrum of genetic diversity, particularly rare variants crucial for adaptive traits. The primary data source, sourced from public databases (Sempéré et al., 2015), might not comprehensively represent the genetic variability within each breed, potentially influencing perceived genetic structures and levels of inbreeding. To address these limitations, future studies should incorporate whole-genome sequencing to provide a more detailed genetic landscape and expand sampling to enhance the robustness of findings. Comparative analysis with cattle breeds under similar pressures in different geographical contexts will further elucidate how selective and environmental pressures shape genetic diversity globally (Freitas et al., 2021).

Our findings underscore the necessity for detailed breeding strategies incorporating genomic tools to enhance traits such as disease resistance and environmental adaptability (Kim et al., 2017b). Linking genetic traits with economic and cultural practices in SSA highlights the importance of preserving genetic diversity for conservation purposes and sustaining local communities’ livelihoods (Vanvanhossou et al., 2021b). This approach supports rural SSA’s cultural heritage and economic stability (Vanvanhossou et al., 2021b).

There is substantial potential for interdisciplinary collaborations among geneticists, breeders, and socio-economists to address the holistic challenges SSA cattle breeds face. Future research could apply genome-wide association studies to pinpoint specific genes linked to desirable characteristics in SSA cattle (Taye et al., 2017). Longitudinal studies tracking genetic changes in these populations will assess the impact of different breeding strategies on genetic diversity and health outcomes.

## 5 Conclusion

This study has yielded pivotal genetic insights into SSA cattle breeds like KUR, NDA, ZBO, and ZFU, which are integral to pastoral and agropastoral systems in the region. Our analysis revealed substantial ROH across these breeds, signaling diverse inbreeding patterns. The NDA breed, in particular, demonstrated the highest inbreeding coefficients, reflecting its history of intense selective breeding. Our findings identified critical ROH islands containing genes such as *RSAD2*, *CMPK2*, and *NOTCH1*, which are linked to immune response and cellular stress mechanisms. These genes likely represent regions of historical selection, conferring adaptive advantages essential for overcoming environmental stresses and diseases prevalent in harsh African environments. Conversely, iHS analysis has revealed recent selective sweeps involving genes like *HIPK1* in KUR cattle, which is known to regulate stress responses and may potentially enhance heat stress resilience. Similarly, *SENP6* and *RIMS1* in NDA cattle are implicated in immune responses and are suggested to play roles in disease resistance. These findings differentiate the roles of genetic markers detected through ROH and iHS, underscoring the breeds’ innate capacities for environmental adaptability and disease resistance. This genetic blueprint provides crucial insights for targeted breeding programs aimed at amplifying these valuable traits. Our work lays the groundwork for informed strategies in livestock management and conservation, ensuring the sustainable development of cattle breeds that are vital to the socioeconomic stability of Sub-Saharan Africa.

## Data Availability

Publicly available datasets were analyzed in this study. This data can be found here: http://widde.toulouse.inra.fr/widde/ under Cattle.
