# Supplementary material for: Genomic insights into adaptation and inbreeding among Sub-Saharan African cattle from pastoral and agropastoral systems
Source: Front Genet. 2024 Jul 25;15:1430291. doi: 10.3389/fgene.2024.1430291 (PMC11306176; doi:10.3389/fgene.2024.1430291)
Supplement: Supplementary file 1 [file Table1.DOCX]

Supplementary Material

Genomic Insights into Adaptation and Inbreeding among Sub-Saharan African Cattle from Pastoral and Agropastoral Systems

**Oludayo M. Akinsola^1^, Abdulraheem A. Musa^2*^, Lalmuansangi^3^, Sanchit P. Singh^3^, Sabyasachi Mukherjee^3^, and Anupama Mukherjee^3^**

^1^Department of Theriogenology and Production, Faculty of Veterinary Medicine, University of Jos, Nigeria

^2^Research Institute for Farm Animal Biology (FBN), 18196 Dummerstorf, Germany

^3^Animal Genetics and Breeding Division, Indian Council of Agricultural Research (ICAR)-National Dairy Research Institute (NDRI), Karnal, Haryana State, India

*** Correspondence:** Abdulraheem A. Musa: [musa@fbn-dummerstorf.de](mailto:musa@fbn-dummerstorf.de)

# Supplementary Figures and Tables

## Supplementary Figures


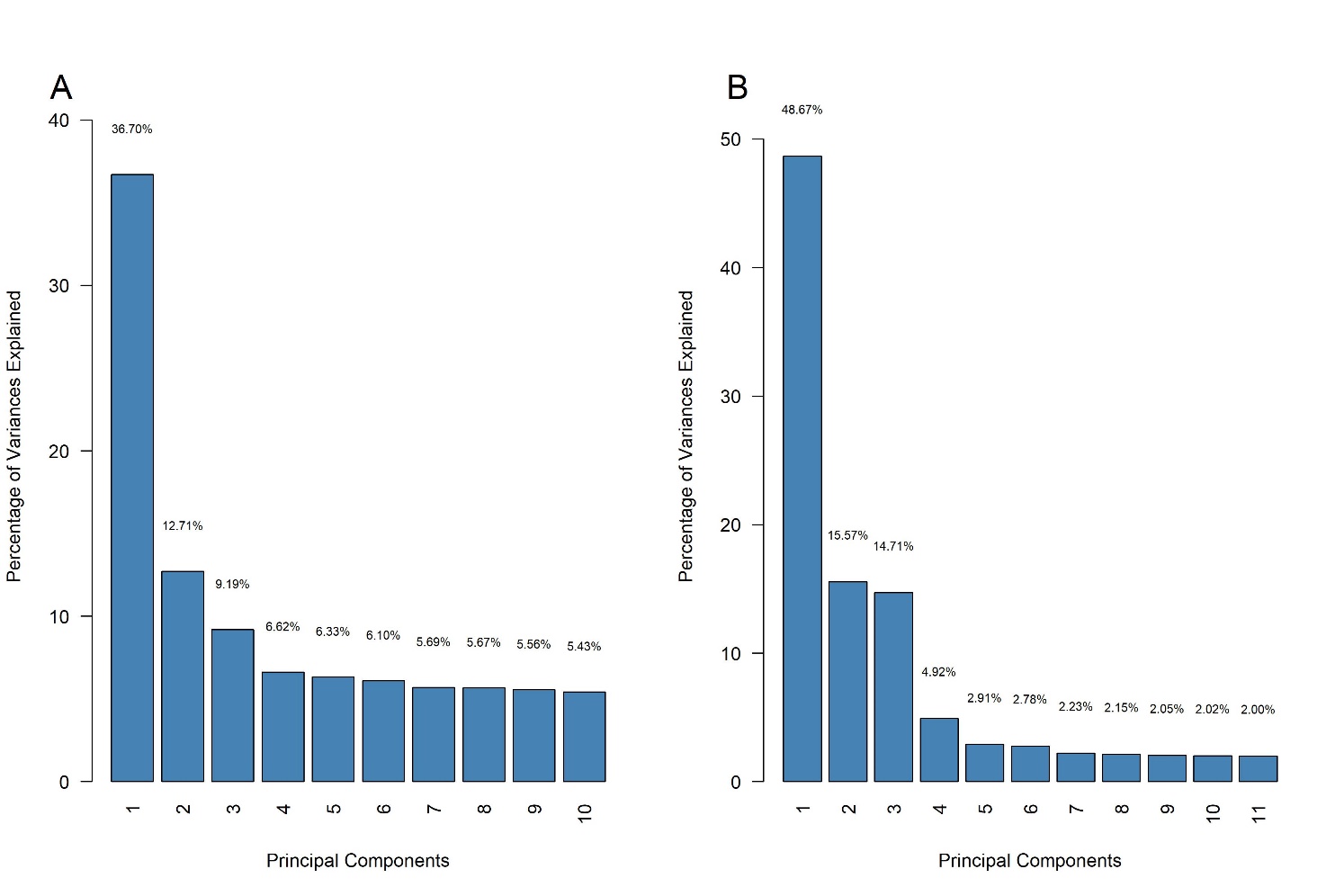


**Figure S1.** Distribution of genetic variance in cattle: Sub-Saharan African breeds (A) and breeds from diverse climatic regions (B).

## Supplementary Tables

| Table S1: Candidate Genomic Regions Identified by ROH Analysis Indicative of Historical Selection in Sub-Saharan African Cattle Breeds | | | | | | | |
| --- | --- | --- | --- | --- | --- | --- | --- |
| **Breed** | **BTA** | **No. of SNPs** | **Start bp** | **End bp** | **No. genes in ROH** | **Gene symbol** | **Product** |
| Ndama | 11 | 16 | 89494181 | 90494181 | 12 | Metazoa_SRP | Metazoan signal recognition particle RNA [Source:RFAM;Acc:RF00017] |
|  |  |  |  |  |  | RNF144A | ring finger protein 144A [Source:VGNC Symbol;Acc:VGNC:34026] |
|  |  |  |  |  |  | RSAD2 | radical S-adenosyl methionine domain containing 2 [Source:VGNC Symbol;Acc:VGNC:34176] |
|  |  |  |  |  |  | CMPK2 | cytidine/uridine monophosphate kinase 2 [Source:VGNC Symbol;Acc:VGNC:27481] |
|  |  |  | 90568648 | 91568648 | 1 | SOX11 | SRY-box transcription factor 11 [Source:VGNC Symbol;Acc:VGNC:106944] |
|  |  |  | 92137746 | 93137746 | 10 | GGTA1 | glycoprotein alpha-galactosyltransferase 1 (inactive) [Source:NCBI gene (formerly Entrezgene);Acc:281780] |
|  |  |  |  |  |  | DAB2IP | DAB2 interacting protein [Source:VGNC Symbol;Acc:VGNC:27866] |
|  |  |  |  |  |  | TTLL11 | tubulin tyrosine ligase like 11 [Source:HGNC Symbol;Acc:HGNC:18113] |
|  |  |  |  |  |  | NDUFA8 | NADH:ubiquinone oxidoreductase subunit A8 [Source:VGNC Symbol;Acc:VGNC:31951] |
|  |  |  |  |  |  | MORN5 | MORN repeat containing 5 [Source:VGNC Symbol;Acc:VGNC:31562] |
|  |  |  |  |  |  | LHX6 | LIM homeobox 6 [Source:VGNC Symbol;Acc:VGNC:30876] |
|  |  |  |  |  |  | RBM18 | RNA binding motif protein 18 [Source:VGNC Symbol;Acc:VGNC:33789] |
|  |  |  | 92945185 | 93945185 | 57 | MRRF | mitochondrial ribosome recycling factor [Source:VGNC Symbol;Acc:VGNC:102827] |
|  |  |  |  |  |  | PTGS1 | prostaglandin-endoperoxide synthase 1 [Source:VGNC Symbol;Acc:VGNC:33511] |
|  |  |  |  |  |  | bta-mir-10175 | bta-mir-10175 [Source:miRBase;Acc:MI0032931] |
|  |  |  |  |  |  | OR1L12 | olfactory receptor family 1 subfamily L member 12 [Source:VGNC Symbol;Acc:VGNC:100585] |
|  |  |  |  |  |  | OR1L8H | olfactory receptor family 1 subfamily L member 8H [Source:VGNC Symbol;Acc:VGNC:100602] |
|  |  |  |  |  |  | OR1L8 | olfactory receptor family 1 subfamily L member 8 [Source:NCBI gene (formerly Entrezgene);Acc:511741] |
|  |  |  |  |  |  | OR1L8C | olfactory receptor family 1 subfamily L member 8C [Source:NCBI gene (formerly Entrezgene);Acc:515482] |
|  |  |  |  |  |  | OR1L8F | olfactory receptor family 1 subfamily L member 8F [Source:NCBI gene (formerly Entrezgene);Acc:100301469] |
|  |  |  |  |  |  | OR1L8E | olfactory receptor family 1 subfamily L member 8E [Source:NCBI gene (formerly Entrezgene);Acc:100337234] |
|  |  |  |  |  |  | OR1L20 | olfactory receptor family 1 subfamily L member 20 [Source:VGNC Symbol;Acc:VGNC:100590] |
|  |  |  |  |  |  | OR1L8D | olfactory receptor family 1 subfamily L member 8D [Source:NCBI gene (formerly Entrezgene);Acc:787415] |
|  |  |  |  |  |  | OR1L21 | olfactory receptor family 1 subfamily L member 21 [Source:VGNC Symbol;Acc:VGNC:100591] |
|  |  |  |  |  |  | OR1L13 | olfactory receptor family 1 subfamily L member 13 [Source:NCBI gene (formerly Entrezgene);Acc:615170] |
|  |  |  |  |  |  | OR1J6 | olfactory receptor family 1 subfamily J member 6 [Source:NCBI gene (formerly Entrezgene);Acc:510731] |
|  |  |  |  |  |  | OR1J32 | olfactory receptor family 1 subfamily J member 32 [Source:NCBI gene (formerly Entrezgene);Acc:119646078] |
|  |  |  |  |  |  | OR1J33 | olfactory receptor family 1 subfamily J member 33 [Source:NCBI gene (formerly Entrezgene);Acc:119646079] |
|  |  |  |  |  |  | OR1J31 | olfactory receptor family 1 subfamily J member 31 [Source:NCBI gene (formerly Entrezgene);Acc:119646077] |
|  |  |  |  |  |  | OR1J4E | olfactory receptor family 1 subfamily J member 4E [Source:NCBI gene (formerly Entrezgene);Acc:100851523] |
|  |  |  |  |  |  | OR1J26 | olfactory receptor family 1 subfamily J member 26 [Source:VGNC Symbol;Acc:VGNC:100574] |
|  |  |  |  |  |  | OR1J1 | olfactory receptor family 1 subfamily J member 1 [Source:HGNC Symbol;Acc:HGNC:8208] |
|  |  |  |  |  |  | OR1J2 | olfactory receptor family 1 subfamily J member 2 [Source:HGNC Symbol;Acc:HGNC:8209] |
|  |  |  |  |  |  | OR1N2 | olfactory receptor family 1 subfamily N member 2 [Source:VGNC Symbol;Acc:VGNC:100605] |
|  |  |  |  |  |  | OR1N1 | olfactory receptor family 1 subfamily N member 1 [Source:NCBI gene (formerly Entrezgene);Acc:107131158] |
|  |  |  |  |  |  | OR1J4B | olfactory receptor family 1 subfamily J member 4B [Source:NCBI gene (formerly Entrezgene);Acc:539172] |
|  |  |  |  |  |  | OR1J4D | olfactory receptor family 1 subfamily J member 4D [Source:NCBI gene (formerly Entrezgene);Acc:119646080] |
|  |  |  |  |  |  | OR1AK3 | olfactory receptor family 1 subfamily AK member 3 [Source:NCBI gene (formerly Entrezgene);Acc:119645953] |
|  |  |  |  |  |  | OR1J28 | olfactory receptor family 1 subfamily J member 28 [Source:VGNC Symbol;Acc:VGNC:100575] |
|  |  |  |  |  |  | OR1Q1 | olfactory receptor family 1 subfamily Q member 1 [Source:VGNC Symbol;Acc:VGNC:100612] |
|  |  |  |  |  |  | OR12K5 | olfactory receptor family 12 subfamily K member 5 [Source:VGNC Symbol;Acc:VGNC:100485] |
|  |  |  |  |  |  | OR1B1 | olfactory receptor family 1 subfamily B member 1 [Source:VGNC Symbol;Acc:VGNC:100551] |
|  |  |  |  |  |  | OR1L22 | olfactory receptor family 1 subfamily L member 22 [Source:VGNC Symbol;Acc:VGNC:100592] |
|  |  |  |  |  |  | OR1L1 | olfactory receptor family 1 subfamily L member 1 [Source:VGNC Symbol;Acc:VGNC:100584] |
|  |  |  |  |  |  | OR1L19 | olfactory receptor family 1 subfamily L member 19 [Source:VGNC Symbol;Acc:VGNC:100589] |
|  |  |  |  |  |  | OR1L4 | olfactory receptor family 1 subfamily L member 4 [Source:NCBI gene (formerly Entrezgene);Acc:504570] |
|  |  |  |  |  |  | OR1L6 | olfactory receptor family 1 subfamily L member 6 [Source:NCBI gene (formerly Entrezgene);Acc:786664] |
|  |  |  |  |  |  | OR1AF3 | olfactory receptor family 1 subfamily AF member 3 [Source:VGNC Symbol;Acc:VGNC:100547] |
|  |  |  |  |  |  | OR1AF1 | olfactory receptor family 1 subfamily AF member 1 [Source:VGNC Symbol;Acc:VGNC:100546] |
|  |  |  |  |  |  | OR1AH3 | olfactory receptor family 1 subfamily AH member 3 [Source:NCBI gene (formerly Entrezgene);Acc:786573] |
|  |  |  |  |  |  | OR1AH6 | olfactory receptor family 1 subfamily AH member 6 [Source:VGNC Symbol;Acc:VGNC:100550] |
|  |  |  | 94058900 | 95058900 | 16 | RC3H2 | ring finger and CCCH-type domains 2 [Source:VGNC Symbol;Acc:VGNC:33819] |
|  |  |  |  |  |  | ZBTB6 | zinc finger and BTB domain containing 6 [Source:VGNC Symbol;Acc:VGNC:37088] |
|  |  |  |  |  |  | ZBTB26 | zinc finger and BTB domain containing 26 [Source:VGNC Symbol;Acc:VGNC:37070] |
|  |  |  |  |  |  | RABGAP1 | RAB GTPase activating protein 1 [Source:VGNC Symbol;Acc:VGNC:106888] |
|  |  |  |  |  |  | STRBP | spermatid perinuclear RNA binding protein [Source:VGNC Symbol;Acc:VGNC:35421] |
|  |  |  |  |  |  | U6 | U6 spliceosomal RNA [Source:RFAM;Acc:RF00026] |
|  |  |  |  |  |  | CRB2 | crumbs cell polarity complex component 2 [Source:VGNC Symbol;Acc:VGNC:27689] |
|  |  |  |  |  |  | DENND1A | DENN domain containing 1A [Source:VGNC Symbol;Acc:VGNC:27993] |
|  |  |  |  |  |  | LHX2 | LIM homeobox 2 [Source:VGNC Symbol;Acc:VGNC:30872] |
|  |  |  |  |  |  | NEK6 | NIMA related kinase 6 [Source:VGNC Symbol;Acc:VGNC:31997] |
|  |  |  |  |  |  | PSMB7 | proteasome 20S subunit beta 7 [Source:VGNC Symbol;Acc:VGNC:33451] |
|  |  |  |  |  |  | ADGRD2 | adhesion G protein-coupled receptor D2 [Source:VGNC Symbol;Acc:VGNC:25660] |
|  |  |  |  |  |  | NR5A1 | nuclear receptor subfamily 5 group A member 1 [Source:VGNC Symbol;Acc:VGNC:32248] |
|  |  |  |  |  |  | NR6A1 | nuclear receptor subfamily 6 group A member 1 [Source:VGNC Symbol;Acc:VGNC:32250] |
|  |  |  | 96277054 | 97277054 | 4 | MAPKAP1 | MAPK associated protein 1 [Source:VGNC Symbol;Acc:VGNC:31228] |
|  |  |  |  |  |  | PBX3 | PBX homeobox 3 [Source:VGNC Symbol;Acc:VGNC:32609] |
|  |  |  |  |  |  | MVB12B | multivesicular body subunit 12B [Source:VGNC Symbol;Acc:VGNC:102829] |
|  |  |  | 96956729 | 97956729 | 12 | LMX1B | LIM homeobox transcription factor 1 beta [Source:VGNC Symbol;Acc:VGNC:102825] |
|  |  |  |  |  |  | ZBTB43 | zinc finger and BTB domain containing 43 [Source:VGNC Symbol;Acc:VGNC:37082] |
|  |  |  |  |  |  | ZBTB34 | zinc finger and BTB domain containing 34 [Source:VGNC Symbol;Acc:VGNC:37074] |
|  |  |  |  |  |  | RALGPS1 | Ral GEF with PH domain and SH3 binding motif 1 [Source:VGNC Symbol;Acc:VGNC:33704] |
|  |  |  |  |  |  | ANGPTL2 | angiopoietin like 2 [Source:VGNC Symbol;Acc:VGNC:25891] |
|  |  |  | 97526258 | 98526258 | 23 | GARNL3 | GTPase activating Rap/RanGAP domain like 3 [Source:VGNC Symbol;Acc:VGNC:29253] |
|  |  |  |  |  |  | SLC2A8 | solute carrier family 2 member 8 [Source:VGNC Symbol;Acc:VGNC:34803] |
|  |  |  |  |  |  | ZNF79 | zinc finger protein 79 [Source:VGNC Symbol;Acc:VGNC:37347] |
|  |  |  |  |  |  | RPL12 | ribosomal protein L12 [Source:NCBI gene (formerly Entrezgene);Acc:404133] |
|  |  |  |  |  |  | LRSAM1 | leucine rich repeat and sterile alpha motif containing 1 [Source:VGNC Symbol;Acc:VGNC:31045] |
|  |  |  |  |  |  | NIBAN2 | niban apoptosis regulator 2 [Source:VGNC Symbol;Acc:VGNC:54427] |
|  |  |  |  |  |  | STXBP1 | syntaxin binding protein 1 [Source:VGNC Symbol;Acc:VGNC:35445] |
|  |  |  |  |  |  | CFAP157 | cilia and flagella associated protein 157 [Source:VGNC Symbol;Acc:VGNC:27238] |
|  |  |  |  |  |  | PTRH1 | peptidyl-tRNA hydrolase 1 homolog [Source:VGNC Symbol;Acc:VGNC:33561] |
|  |  |  |  |  |  | TTC16 | tetratricopeptide repeat domain 16 [Source:VGNC Symbol;Acc:VGNC:36456] |
|  |  |  |  |  |  | TOR2A | torsin family 2 member A [Source:VGNC Symbol;Acc:VGNC:49983] |
|  |  |  |  |  |  | SH2D3C | SH2 domain containing 3C [Source:VGNC Symbol;Acc:VGNC:34559] |
|  |  |  |  |  |  | CDK9 | cyclin dependent kinase 9 [Source:VGNC Symbol;Acc:VGNC:27136] |
|  |  |  |  |  |  | FPGS | folylpolyglutamate synthase [Source:VGNC Symbol;Acc:VGNC:55047] |
|  |  |  |  |  |  | ENG | endoglin [Source:VGNC Symbol;Acc:VGNC:102821] |
|  |  |  | 99046782 | 100046782 | 38 | ODF2 | outer dense fiber of sperm tails 2 [Source:VGNC Symbol;Acc:VGNC:32401] |
|  |  |  |  |  |  | GLE1 | GLE1 RNA export mediator [Source:VGNC Symbol;Acc:VGNC:29397] |
|  |  |  |  |  |  | SPTAN1 | spectrin alpha, non-erythrocytic 1 [Source:VGNC Symbol;Acc:VGNC:35251] |
|  |  |  |  |  |  | DYNC2I2 | dynein 2 intermediate chain 2 [Source:VGNC Symbol;Acc:VGNC:36893] |
|  |  |  |  |  |  | SET | SET nuclear proto-onco [Source:NCBI gene (formerly Entrezgene);Acc:538444] |
|  |  |  |  |  |  | PKN3 | protein kinase N3 [Source:VGNC Symbol;Acc:VGNC:32947] |
|  |  |  |  |  |  | ZDHHC12 | zinc finger DHHC-type palmitoyltransferase 12 [Source:VGNC Symbol;Acc:VGNC:37127] |
|  |  |  |  |  |  | ZER1 | zyg-11 related cell cycle regulator [Source:VGNC Symbol;Acc:VGNC:37148] |
|  |  |  |  |  |  | TBC1D13 | TBC1 domain family member 13 [Source:VGNC Symbol;Acc:VGNC:35627] |
|  |  |  |  |  |  | ENDOG | endonuclease G [Source:VGNC Symbol;Acc:VGNC:28491] |
|  |  |  |  |  |  | SPOUT1 | SPOUT domain containing methyltransferase 1 [Source:VGNC Symbol;Acc:VGNC:35231] |
|  |  |  |  |  |  | KYAT1 | kynurenine aminotransferase 1 [Source:NCBI gene (formerly Entrezgene);Acc:528582] |
|  |  |  |  |  |  | LRRC8A | leucine rich repeat containing 8 VRAC subunit A [Source:VGNC Symbol;Acc:VGNC:31029] |
|  |  |  |  |  |  | PHYHD1 | phytanoyl-CoA dioxygenase domain containing 1 [Source:NCBI gene (formerly Entrezgene);Acc:540828] |
|  |  |  |  |  |  | DOLK | dolichol kinase [Source:VGNC Symbol;Acc:VGNC:28170] |
|  |  |  |  |  |  | NUP188 | nucleoporin 188 [Source:VGNC Symbol;Acc:VGNC:32354] |
|  |  |  |  |  |  | SH3GLB2 | SH3 domain containing GRB2 like, endophilin B2 [Source:VGNC Symbol;Acc:VGNC:34576] |
|  |  |  |  |  |  | MIGA2 | mitoguardin 2 [Source:VGNC Symbol;Acc:VGNC:31474] |
|  |  |  |  |  |  | DOLPP1 | dolichyldiphosphatase 1 [Source:VGNC Symbol;Acc:VGNC:28171] |
|  |  |  |  |  |  | CRAT | carnitine O-acetyltransferase [Source:VGNC Symbol;Acc:VGNC:27687] |
|  |  |  |  |  |  | PTPA | protein phosphatase 2 phosphatase activator [Source:VGNC Symbol;Acc:VGNC:33526] |
|  |  |  |  |  |  | bta-mir-12056 | bta-mir-12056 [Source:miRBase;Acc:MI0038489] |
|  |  |  |  |  |  | IER5L | immediate early response 5 like [Source:VGNC Symbol;Acc:VGNC:30041] |
|  |  |  |  |  |  | C11H9orf50 | chromosome 11 C9orf50 homolog [Source:VGNC Symbol;Acc:VGNC:52622] |
|  |  |  |  |  |  | NTMT1 | N-terminal Xaa-Pro-Lys N-methyltransferase 1 [Source:VGNC Symbol;Acc:VGNC:32303] |
|  |  |  |  |  |  | ASB6 | ankyrin repeat and SOCS box containing 6 [Source:VGNC Symbol;Acc:VGNC:26199] |
|  |  |  | 99783991 | 100783991 | 21 | PRRX2 | paired related homeobox 2 [Source:VGNC Symbol;Acc:VGNC:58400] |
|  |  |  |  |  |  | PTGES | prostaglandin E synthase [Source:VGNC Symbol;Acc:VGNC:33505] |
|  |  |  |  |  |  | TOR1B | torsin family 1 member B [Source:VGNC Symbol;Acc:VGNC:54504] |
|  |  |  |  |  |  | TOR1A | torsin family 1 member A [Source:VGNC Symbol;Acc:VGNC:36223] |
|  |  |  |  |  |  | C11H9orf78 | chromosome 11 C9orf78 homolog [Source:VGNC Symbol;Acc:VGNC:52623] |
|  |  |  |  |  |  | USP20 | ubiquitin specific peptidase 20 [Source:VGNC Symbol;Acc:VGNC:36715] |
|  |  |  |  |  |  | FNBP1 | formin binding protein 1 [Source:VGNC Symbol;Acc:VGNC:29059] |
|  |  |  |  |  |  | GPR107 | G protein-coupled receptor 107 [Source:VGNC Symbol;Acc:VGNC:29544] |
|  |  |  |  |  |  | NCS1 | neuronal calcium sensor 1 [Source:VGNC Symbol;Acc:VGNC:31928] |
|  |  |  |  |  |  | HMCN2 | hemicentin 2 [Source:HGNC Symbol;Acc:HGNC:21293] |
|  |  |  |  |  |  | ASS1 | argininosuccinate synthase 1 [Source:VGNC Symbol;Acc:VGNC:26224] |
|  |  |  | 101375858 | 102375858 | 19 | NUP214 | nucleoporin 214 [Source:VGNC Symbol;Acc:VGNC:32358] |
|  |  |  |  |  |  | FAM78A | family with sequence similarity 78 member A [Source:VGNC Symbol;Acc:VGNC:28829] |
|  |  |  |  |  |  | PLPP7 | phospholipid phosphatase 7 (inactive) [Source:HGNC Symbol;Acc:HGNC:28174] |
|  |  |  |  |  |  | PRRC2B | proline rich coiled-coil 2B [Source:VGNC Symbol;Acc:VGNC:33405] |
|  |  |  |  |  |  | SNORD62 | Small nucleolar RNA SNORD62 [Source:RFAM;Acc:RF00153] |
|  |  |  |  |  |  | POMT1 | protein O-mannosyltransferase 1 [Source:VGNC Symbol;Acc:VGNC:33158] |
|  |  |  |  |  |  | UCK1 | uridine-cytidine kinase 1 [Source:VGNC Symbol;Acc:VGNC:36634] |
|  |  |  |  |  |  | PRRT1B | proline rich transmembrane protein 1B [Source:VGNC Symbol;Acc:VGNC:106879] |
|  |  |  |  |  |  | RAPGEF1 | Rap guanine nucleotide exchange factor 1 [Source:VGNC Symbol;Acc:VGNC:33722] |
|  |  |  |  |  |  | MED27 | mediator complex subunit 27 [Source:VGNC Symbol;Acc:VGNC:31363] |
|  |  |  |  |  |  | NTNG2 | netrin G2 [Source:VGNC Symbol;Acc:VGNC:32308] |
|  |  |  |  |  |  | SETX | senataxin [Source:VGNC Symbol;Acc:VGNC:34501] |
|  |  |  | 101653173 | 102653173 | 14 | TTF1 | transcription termination factor 1 [Source:VGNC Symbol;Acc:VGNC:36483] |
|  |  |  |  |  |  | CFAP77 | cilia and flagella associated protein 77 [Source:VGNC Symbol;Acc:VGNC:59210] |
|  |  |  |  |  |  | DDX31 | DEAD-box helicase 31 [Source:VGNC Symbol;Acc:VGNC:27963] |
|  |  |  |  |  |  | BARHL1 | BarH like homeobox 1 [Source:VGNC Symbol;Acc:VGNC:26421] |
|  |  |  | 102958444 | 103958444 | 39 | TSC1 | TSC complex subunit 1 [Source:VGNC Symbol;Acc:VGNC:36407] |
|  |  |  |  |  |  | GFI1B | growth factor independent 1B transcriptional repressor [Source:VGNC Symbol;Acc:VGNC:29326] |
|  |  |  |  |  |  | GTF3C5 | ral transcription factor IIIC subunit 5 [Source:VGNC Symbol;Acc:VGNC:29706] |
|  |  |  |  |  |  | CEL | carboxyl ester lipase [Source:VGNC Symbol;Acc:VGNC:27165] |
|  |  |  |  |  |  | RALGDS | ral guanine nucleotide dissociation stimulator [Source:NCBI gene (formerly Entrezgene);Acc:513723] |
|  |  |  |  |  |  | GBGT1 | globoside alpha-1,3-N-acetylgalactosaminyltransferase 1 [Source:VGNC Symbol;Acc:VGNC:59217] |
|  |  |  |  |  |  | GLT6D1 | glycosyltransferase 6 domain containing 1 [Source:VGNC Symbol;Acc:VGNC:29422] |
|  |  |  |  |  |  | LCN9 | lipocalin 9 [Source:VGNC Symbol;Acc:VGNC:30815] |
|  |  |  |  |  |  | KCNT1 | potassium sodium-activated channel subfamily T member 1 [Source:VGNC Symbol;Acc:VGNC:30494] |
|  |  |  |  |  |  | CAMSAP1 | calmodulin regulated spectrin associated protein 1 [Source:VGNC Symbol;Acc:VGNC:26730] |
|  |  |  |  |  |  | UBAC1 | UBA domain containing 1 [Source:VGNC Symbol;Acc:VGNC:36567] |
|  |  |  |  |  |  | NACC2 | NACC family member 2 [Source:VGNC Symbol;Acc:VGNC:31862] |
|  |  |  |  |  |  | TMEM250 | transmembrane protein 250 [Source:VGNC Symbol;Acc:VGNC:36059] |
|  |  |  |  |  |  | LHX3 | LIM homeobox 3 [Source:VGNC Symbol;Acc:VGNC:30873] |
|  |  |  |  |  |  | QSOX2 | quiescin sulfhydryl oxidase 2 [Source:VGNC Symbol;Acc:VGNC:33606] |
|  |  |  |  |  |  | GPSM1 | G protein signaling modulator 1 [Source:VGNC Symbol;Acc:VGNC:29614] |
|  |  |  |  |  |  | DNLZ | DNL-type zinc finger [Source:HGNC Symbol;Acc:HGNC:33879] |
|  |  |  |  |  |  | CARD9 | caspase recruitment domain family member 9 [Source:VGNC Symbol;Acc:VGNC:26764] |
|  |  |  |  |  |  | SNAPC4 | small nuclear RNA activating complex polypeptide 4 [Source:VGNC Symbol;Acc:VGNC:58415] |
|  |  |  |  |  |  | ENTR1 | endosome associated trafficking regulator 1 [Source:VGNC Symbol;Acc:VGNC:54703] |
|  |  |  |  |  |  | PMPCA | peptidase, mitochondrial processing subunit alpha [Source:VGNC Symbol;Acc:VGNC:33075] |
|  |  |  |  |  |  | INPP5E | inositol polyphosphate-5-phosphatase E [Source:VGNC Symbol;Acc:VGNC:30213] |
|  |  |  |  |  |  | SEC16A | SEC16 homolog A, endoplasmic reticulum export factor [Source:VGNC Symbol;Acc:VGNC:34406] |
|  |  |  |  |  |  | NOTCH1 | notch receptor 1 [Source:VGNC Symbol;Acc:VGNC:32177] |
|  |  |  | 103591220 | 104591220 | 54 | EGFL7 | EGF like domain multiple 7 [Source:VGNC Symbol;Acc:VGNC:59214] |
|  |  |  |  |  |  | bta-mir-126 | bta-mir-126 [Source:miRBase;Acc:MI0004754] |
|  |  |  |  |  |  | AGPAT2 | 1-acylglycerol-3-phosphate O-acyltransferase 2 [Source:VGNC Symbol;Acc:VGNC:25735] |
|  |  |  |  |  |  | DIPK1B | divergent protein kinase domain 1B [Source:VGNC Symbol;Acc:VGNC:28825] |
|  |  |  |  |  |  | ABO | ABO, alpha 1-3-N-acetylgalactosaminyltransferase and alpha 1-3-galactosyltransferase [Source:VGNC Symbol;Acc:VGNC:25510] |
|  |  |  |  |  |  | SURF6 | surfeit 6 [Source:VGNC Symbol;Acc:VGNC:35485] |
|  |  |  |  |  |  | MED22 | mediator complex subunit 22 [Source:VGNC Symbol;Acc:VGNC:31359] |
|  |  |  |  |  |  | RPL7A | ribosomal protein L7a [Source:VGNC Symbol;Acc:VGNC:49957] |
|  |  |  |  |  |  | SNORD24 | Small nucleolar RNA SNORD24 [Source:RFAM;Acc:RF00069] |
|  |  |  |  |  |  | SURF1 | SURF1 cytochrome c oxidase assembly factor [Source:VGNC Symbol;Acc:VGNC:106961] |
|  |  |  |  |  |  | SNORD36 | Small nucleolar RNA SNORD36 [Source:RFAM;Acc:RF00049] |
|  |  |  |  |  |  | SURF2 | surfeit 2 [Source:VGNC Symbol;Acc:VGNC:35484] |
|  |  |  |  |  |  | SURF4 | surfeit 4 [Source:VGNC Symbol;Acc:VGNC:106962] |
|  |  |  |  |  |  | STKLD1 | serine/threonine kinase like domain containing 1 [Source:VGNC Symbol;Acc:VGNC:35403] |
|  |  |  |  |  |  | REXO4 | REX4 homolog, 3'-5' exonuclease [Source:VGNC Symbol;Acc:VGNC:33885] |
|  |  |  |  |  |  | ADAMTS13 | ADAM metallopeptidase with thrombospondin type 1 motif 13 [Source:VGNC Symbol;Acc:VGNC:25617] |
|  |  |  |  |  |  | CACFD1 | calcium channel flower domain containing 1 [Source:VGNC Symbol;Acc:VGNC:26670] |
|  |  |  |  |  |  | SLC2A6 | solute carrier family 2 member 6 [Source:VGNC Symbol;Acc:VGNC:34802] |
|  |  |  |  |  |  | MYMK | myomaker, myoblast fusion factor [Source:VGNC Symbol;Acc:VGNC:31810] |
|  |  |  |  |  |  | ADAMTSL2 | ADAMTS like 2 [Source:VGNC Symbol;Acc:VGNC:25631] |
|  |  |  |  |  |  | FAM163B | family with sequence similarity 163 member B [Source:NCBI gene (formerly Entrezgene);Acc:615192] |
|  |  |  |  |  |  | DBH | dopamine beta-hydroxylase [Source:VGNC Symbol;Acc:VGNC:27888] |
|  |  |  |  |  |  | SARDH | sarcosine dehydrogenase [Source:VGNC Symbol;Acc:VGNC:34289] |
|  |  |  |  |  |  | VAV2 | vav guanine nucleotide exchange factor 2 [Source:VGNC Symbol;Acc:VGNC:36773] |
|  | 28 | 5 | 42381677 | 43381677 | 7 | PTPN20 | protein tyrosine phosphatase non-receptor type 20 [Source:HGNC Symbol;Acc:HGNC:23423] |
|  |  |  |  |  |  | FRMPD2 | FERM and PDZ domain containing 2 [Source:VGNC Symbol;Acc:VGNC:50131] |
|  |  |  |  |  |  | MAPK8 | mitogen-activated protein kinase 8 [Source:VGNC Symbol;Acc:VGNC:31223] |
|  |  |  |  |  |  | ARHGAP22 | Rho GTPase activating protein 22 [Source:VGNC Symbol;Acc:VGNC:26081] |
|  |  |  |  |  |  | WDFY4 | WDFY family member 4 [Source:VGNC Symbol;Acc:VGNC:55999] |
|  |  |  |  |  |  | LRRC18 | leucine rich repeat containing 18 [Source:VGNC Symbol;Acc:VGNC:31002] |
|  |  |  |  |  |  | VSTM4 | V-set and transmembrane domain containing 4 [Source:VGNC Symbol;Acc:VGNC:54511] |
|  |  |  | 43020975 | 44020975 | 16 | FAM170B | family with sequence similarity 170 member B [Source:VGNC Symbol;Acc:VGNC:28755] |
|  |  |  |  |  |  | C28H10orf71 | chromosome 28 C10orf71 homolog [Source:VGNC Symbol;Acc:VGNC:52696] |
|  |  |  |  |  |  | DRGX | dorsal root ganglia homeobox [Source:VGNC Symbol;Acc:VGNC:28210] |
|  |  |  |  |  |  | ERCC6 | ERCC excision repair 6, chromatin remodeling factor [Source:VGNC Symbol;Acc:VGNC:28572] |
|  |  |  |  |  |  | SLC18A3 | solute carrier family 18 member A3 [Source:VGNC Symbol;Acc:VGNC:34707] |
|  |  |  |  |  |  | CHAT | choline O-acetyltransferase [Source:VGNC Symbol;Acc:VGNC:27269] |
|  |  |  |  |  |  | C28H10orf53 | chromosome 28 C10orf53 homolog [Source:VGNC Symbol;Acc:VGNC:52695] |
|  |  |  |  |  |  | OGDHL | oxoglutarate dehydrogenase L [Source:VGNC Symbol;Acc:VGNC:32407] |
|  |  |  |  |  |  | PARG | poly(ADP-ribose) glycohydrolase [Source:NCBI gene (formerly Entrezgene);Acc:281377] |
|  |  |  |  |  |  | TIMM23 | translocase of inner mitochondrial membrane 23 homolog (yeast) [Source:NCBI gene (formerly Entrezgene);Acc:509841] |
|  |  |  | 43653620 | 44653620 | 26 | SNORA74 | Small nucleolar RNA SNORA74 [Source:RFAM;Acc:RF00090] |
|  |  |  |  |  |  | NCOA4 | nuclear receptor coactivator 4 [Source:VGNC Symbol;Acc:VGNC:31922] |
|  |  |  |  |  |  | MSMB | microseminoprotein beta [Source:VGNC Symbol;Acc:VGNC:31700] |
|  |  |  |  |  |  | WASHC2A | WASH complex subunit 2A [Source:NCBI gene (formerly Entrezgene);Acc:513740] |
|  |  |  |  |  |  | ZFAND4 | zinc finger AN1-type containing 4 [Source:VGNC Symbol;Acc:VGNC:37152] |
|  |  |  |  |  |  | MARCHF8 | membrane associated ring-CH-type finger 8 [Source:VGNC Symbol;Acc:VGNC:55990] |
|  |  |  |  |  |  | ALOX5 | arachidonate 5-lipoxygenase [Source:VGNC Symbol;Acc:VGNC:25844] |
|  |  |  |  |  |  | OR13A1 | olfactory receptor family 13 subfamily A member 1 [Source:VGNC Symbol;Acc:VGNC:100486] |
|  |  |  |  |  |  | OR6D16 | olfactory receptor family 6 subfamily D member 16 [Source:VGNC Symbol;Acc:VGNC:101767] |
|  |  |  |  |  |  | ZNF22 | zinc finger protein 22 [Source:NCBI gene (formerly Entrezgene);Acc:768051] |
|  |  |  |  |  |  | DEPP1 | DEPP autophagy regulator 1 [Source:VGNC Symbol;Acc:VGNC:49544] |
|  |  |  |  |  |  | RASSF4 | Ras association domain family member 4 [Source:VGNC Symbol;Acc:VGNC:33760] |
|  |  |  |  |  |  | TMEM72 | transmembrane protein 72 [Source:VGNC Symbol;Acc:VGNC:36110] |
|  |  |  | 44762079 | 45762079 | 8 | CXCL12 | C-X-C motif chemokine ligand 12 [Source:VGNC Symbol;Acc:VGNC:27848] |
|  |  |  |  |  |  | ZNF32 | zinc finger protein 32 [Source:VGNC Symbol;Acc:VGNC:37247] |
|  |  |  |  |  |  | TFAM | transcription factor A, mitochondrial [Source:VGNC Symbol;Acc:VGNC:35775] |
|  |  |  |  |  |  | AGT | angiotensinogen [Source:VGNC Symbol;Acc:VGNC:103718] |
|  |  |  |  |  |  | COG2 | component of oligomeric golgi complex 2 [Source:VGNC Symbol;Acc:VGNC:27547] |
| Zebu-Bororo | 5 | 5 | 110436150 | 111436150 | 35 | NPTXR | neuronal pentraxin receptor [Source:VGNC Symbol;Acc:VGNC:32220] |
|  |  |  |  |  |  | CBX6 | chromobox 6 [Source:VGNC Symbol;Acc:VGNC:26819] |
|  |  |  |  |  |  | bta-mir-2285m-4 | bta-mir-2285m-4 [Source:miRBase;Acc:MI0022306] |
|  |  |  |  |  |  | APOBEC3H | apolipoprotein B mRNA editing enzyme catalytic subunit 3H [Source:VGNC Symbol;Acc:VGNC:108124] |
|  |  |  |  |  |  | CBX7 | chromobox 7 [Source:VGNC Symbol;Acc:VGNC:26820] |
|  |  |  |  |  |  | PDGFB | platelet derived growth factor subunit B [Source:VGNC Symbol;Acc:VGNC:32690] |
|  |  |  |  |  |  | RPL3 | ribosomal protein L3 [Source:VGNC Symbol;Acc:VGNC:101451] |
|  |  |  |  |  |  | SNORD83B | small nucleolar RNA, C/D box 83B [Source:HGNC Symbol;Acc:HGNC:17132] |
|  |  |  |  |  |  | bta-mir-2440 | bta-mir-2440 [Source:miRBase;Acc:MI0011493] |
|  |  |  |  |  |  | SNORD83A | small nucleolar RNA, C/D box 83A [Source:HGNC Symbol;Acc:HGNC:17131] |
|  |  |  |  |  |  | SNORD43 | small nucleolar RNA, C/D box 43 [Source:HGNC Symbol;Acc:HGNC:10182] |
|  |  |  |  |  |  | SYNGR1 | synaptogyrin 1 [Source:VGNC Symbol;Acc:VGNC:35524] |
|  |  |  |  |  |  | TAB1 | TGF-beta activated kinase 1 (MAP3K7) binding protein 1 [Source:VGNC Symbol;Acc:VGNC:58447] |
|  |  |  |  |  |  | MGAT3 | beta-1,4-mannosyl-glycoprotein 4-beta-N-acetylglucosaminyltransferase [Source:VGNC Symbol;Acc:VGNC:96698] |
|  |  |  |  |  |  | MIEF1 | mitochondrial elongation factor 1 [Source:VGNC Symbol;Acc:VGNC:31467] |
|  |  |  |  |  |  | ATF4 | activating transcription factor 4 [Source:VGNC Symbol;Acc:VGNC:26242] |
|  |  |  |  |  |  | RPS19BP1 | ribosomal protein S19 binding protein 1 [Source:VGNC Symbol;Acc:VGNC:34132] |
|  |  |  |  |  |  | CACNA1I | calcium voltage-gated channel subunit alpha1 I [Source:VGNC Symbol;Acc:VGNC:55869] |
|  |  |  |  |  |  | ENTHD1 | ENTH domain containing 1 [Source:VGNC Symbol;Acc:VGNC:28508] |
|  |  |  |  |  |  | GRAP2 | GRB2 related adaptor protein 2 [Source:VGNC Symbol;Acc:VGNC:29627] |
|  |  |  |  |  |  | FAM83F | family with sequence similarity 83 member F [Source:VGNC Symbol;Acc:VGNC:28837] |
|  |  |  | 110436150 | 111436150 | 35 | TNRC6B | trinucleotide repeat containing adaptor 6B [Source:VGNC Symbol;Acc:VGNC:36205] |
|  |  |  | 111358987 | 112358987 | 22 | ADSL | adenylosuccinate lyase [Source:VGNC Symbol;Acc:VGNC:58439] |
|  |  |  |  |  |  | SGSM3 | small G protein signaling modulator 3 [Source:VGNC Symbol;Acc:VGNC:34552] |
|  |  |  |  |  |  | MRTFA | myocardin related transcription factor A [Source:VGNC Symbol;Acc:VGNC:55393] |
|  |  |  |  |  |  | bta-mir-2439 | bta-mir-2439 [Source:miRBase;Acc:MI0011492] |
|  |  |  |  |  |  | MCHR1 | melanin concentrating hormone receptor 1 [Source:VGNC Symbol;Acc:VGNC:31305] |
|  |  |  |  |  |  | SLC25A17 | solute carrier family 25 member 17 [Source:VGNC Symbol;Acc:VGNC:34746] |
|  |  |  |  |  |  | ST13 | ST13 Hsp70 interacting protein [Source:VGNC Symbol;Acc:VGNC:58446] |
|  |  |  |  |  |  | XPNPEP3 | X-prolyl aminopeptidase 3 [Source:VGNC Symbol;Acc:VGNC:36996] |
|  |  |  |  |  |  | RBX1 |  |
|  |  |  |  |  |  | bta-mir-1281 | bta-mir-1281 [Source:miRBase;Acc:MI0010466] |
|  |  |  |  |  |  | EP300 | E1A binding protein p300 [Source:VGNC Symbol;Acc:VGNC:57349] |
|  |  |  | 111994968 | 112994968 | 42 | L3MBTL2 | L3MBTL histone methyl-lysine binding protein 2 [Source:VGNC Symbol;Acc:VGNC:30763] |
|  |  |  |  |  |  | bta-mir-2441 | bta-mir-2441 [Source:miRBase;Acc:MI0011494] |
|  |  |  |  |  |  | CHADL | chondroadherin like [Source:VGNC Symbol;Acc:VGNC:27265] |
|  |  |  |  |  |  | RANGAP1 | Ran GTPase activating protein 1 [Source:VGNC Symbol;Acc:VGNC:33713] |
|  |  |  |  |  |  | ZC3H7B | zinc finger CCCH-type containing 7B [Source:VGNC Symbol;Acc:VGNC:37109] |
|  |  |  |  |  |  | TEF | TEF transcription factor, PAR bZIP family member [Source:VGNC Symbol;Acc:VGNC:35734] |
|  |  |  |  |  |  | TOB2 | transducer of ERBB2, 2 [Source:VGNC Symbol;Acc:VGNC:36213] |
|  |  |  |  |  |  | PHF5A | PHD finger protein 5A [Source:VGNC Symbol;Acc:VGNC:32826] |
|  |  |  |  |  |  | ACO2 | aconitase 2 [Source:VGNC Symbol;Acc:VGNC:25546] |
|  |  |  |  |  |  | POLR3H | RNA polymerase III subunit H [Source:VGNC Symbol;Acc:VGNC:33152] |
|  |  |  |  |  |  | CSDC2 | cold shock domain containing C2 [Source:VGNC Symbol;Acc:VGNC:27750] |
|  |  |  |  |  |  | PMM1 | phosphomannomutase 1 [Source:VGNC Symbol;Acc:VGNC:33072] |
|  |  |  |  |  |  | XRCC6 | X-ray repair cross complementing 6 [Source:VGNC Symbol;Acc:VGNC:37007] |
|  |  |  |  |  |  | DESI1 | desumoylating isopeptidase 1 [Source:VGNC Symbol;Acc:VGNC:28014] |
|  |  |  |  |  |  | SNU13 | small nuclear ribonucleoprotein 13 [Source:VGNC Symbol;Acc:VGNC:35087] |
|  |  |  |  |  |  | MEI1 | meiotic double-stranded break formation protein 1 [Source:VGNC Symbol;Acc:VGNC:31378] |
|  |  |  |  |  |  | SNORA70 | Small nucleolar RNA SNORA70 [Source:RFAM;Acc:RF00156] |
|  |  |  |  |  |  | CCDC134 | coiled-coil domain containing 134 [Source:VGNC Symbol;Acc:VGNC:26847] |
|  |  |  |  |  |  | SREBF2 | sterol regulatory element binding transcription factor 2 [Source:VGNC Symbol;Acc:VGNC:35275] |
|  |  |  |  |  |  | bta-mir-33a | bta-mir-33a [Source:miRBase;Acc:MI0009807] |
|  |  |  |  |  |  | SHISA8 | shisa family member 8 [Source:VGNC Symbol;Acc:VGNC:106926] |
|  |  |  |  |  |  | TNFRSF13C | TNF receptor superfamily member 13C [Source:VGNC Symbol;Acc:VGNC:57365] |
|  |  |  |  |  |  | CENPM | centromere protein M [Source:VGNC Symbol;Acc:VGNC:27181] |
|  |  |  |  |  |  | SMIM45 | small integral membrane protein 45 [Source:HGNC Symbol;Acc:HGNC:27930] |
|  |  |  |  |  |  | SEPTIN3 | septin 3 [Source:VGNC Symbol;Acc:VGNC:34455] |
|  |  |  |  |  |  | WBP2NL | WBP2 N-terminal like [Source:VGNC Symbol;Acc:VGNC:36873] |
|  |  |  |  |  |  | NAGA | alpha-N-acetylgalactosaminidase [Source:VGNC Symbol;Acc:VGNC:31867] |
|  |  |  |  |  |  | PHETA2 | PH domain containing endocytic trafficking adaptor 2 [Source:VGNC Symbol;Acc:VGNC:28715] |
|  |  |  |  |  |  | NDUFA6 | NADH:ubiquinone oxidoreductase subunit A6 [Source:VGNC Symbol;Acc:VGNC:31950] |
|  |  |  |  |  |  | CYP2D43 | cytochrome P450 family 2 subfamily D member 43 [Source:VGNC Symbol;Acc:VGNC:110255] |
|  |  |  |  |  |  | CYP2D6 | cytochrome P450 family 2 subfamily D member 6 [Source:VGNC Symbol;Acc:VGNC:111838] |
|  |  |  |  |  |  | TCF20 | transcription factor 20 [Source:VGNC Symbol;Acc:VGNC:35689] |
|  |  |  |  |  |  | bta-mir-2442 | bta-mir-2442 [Source:miRBase;Acc:MI0011495] |
|  |  |  |  |  |  | NFAM1 | NFAT activating protein with ITAM motif 1 [Source:VGNC Symbol;Acc:VGNC:32027] |
|  |  |  |  |  |  | SERHL2 | serine hydrolase like 2 [Source:VGNC Symbol;Acc:VGNC:58453] |
|  |  |  |  |  |  | RRP7 | ribosomal RNA processing 7 homolog [Source:VGNC Symbol;Acc:VGNC:96875] |
|  |  |  |  |  |  | POLDIP3 | DNA polymerase delta interacting protein 3 [Source:VGNC Symbol;Acc:VGNC:33117] |
|  |  |  |  |  |  | U12 | U12 minor spliceosomal RNA [Source:RFAM;Acc:RF00007] |
|  |  |  |  |  |  | CYB5R3 | cytochrome b5 reductase 3 [Source:VGNC Symbol;Acc:VGNC:49054] |
|  |  |  |  |  |  | A4GALT | alpha 1,4-galactosyltransferase (P blood group) [Source:HGNC Symbol;Acc:HGNC:18149] |
|  |  |  |  |  |  | ARFGAP3 | ADP ribosylation factor GTPase activating protein 3 [Source:VGNC Symbol;Acc:VGNC:26063] |
|  |  |  |  |  |  | PACSIN2 | protein kinase C and casein kinase substrate in neurons 2 [Source:VGNC Symbol;Acc:VGNC:32543] |
|  |  |  | 113585555 | 114585555 | 18 | TTLL1 | TTL family tubulin polyglutamylase complex subunit L1 [Source:VGNC Symbol;Acc:VGNC:36489] |
|  |  |  |  |  |  | MCAT | malonyl-CoA-acyl carrier protein transacylase [Source:VGNC Symbol;Acc:VGNC:31297] |
|  |  |  |  |  |  | TSPO | translocator protein [Source:VGNC Symbol;Acc:VGNC:49066] |
|  |  |  |  |  |  | TTLL12 | tubulin tyrosine ligase like 12 [Source:VGNC Symbol;Acc:VGNC:36491] |
|  |  |  |  |  |  | SCUBE1 | signal peptide, CUB domain and EGF like domain containing 1 [Source:VGNC Symbol;Acc:VGNC:34371] |
|  |  |  |  |  |  | MPPED1 | metallophosphoesterase domain containing 1 [Source:VGNC Symbol;Acc:VGNC:106826] |
|  |  |  |  |  |  | EFCAB6 | EF-hand calcium binding domain 6 [Source:VGNC Symbol;Acc:VGNC:28346] |
|  |  |  |  |  |  | SULT4A1 | sulfotransferase family 4A member 1 [Source:VGNC Symbol;Acc:VGNC:35469] |
|  |  |  |  |  |  | PNPLA5 | patatin like phospholipase domain containing 5 [Source:HGNC Symbol;Acc:HGNC:24888] |

| Table S2: Candidate Genomic Regions Identified by iHS Indicating Selective Sweeps in Sub-Saharan African Cattle | | | | | | |
| --- | --- | --- | --- | --- | --- | --- |
| **Breed** | **BTA** | **Number of SNPs** | **Start bp** | **End bp** | **Gene symbol** | **Product** |
| Kuri | 3 | 37 | 29400000 | 31400000 | HIPK1 | homeodomain interacting protein kinase 1 [Source:VGNC Symbol;Acc:VGNC:29859] |
|  |  |  |  |  | DCLRE1B | DNA cross-link repair 1B [Source:VGNC Symbol;Acc:VGNC:27917] |
|  |  |  |  |  | AP4B1 | adaptor related protein complex 4 subunit beta 1 [Source:VGNC Symbol;Acc:VGNC:25991] |
|  |  |  |  |  | BCL2L15 | BCL2 like 15 [Source:VGNC Symbol;Acc:VGNC:26449] |
|  |  |  |  |  | PTPN22 | protein tyrosine phosphatase non-receptor type 22 [Source:VGNC Symbol;Acc:VGNC:33536] |
|  |  |  |  |  | RSBN1 | round spermatid basic protein 1 [Source:VGNC Symbol;Acc:VGNC:34177] |
|  |  |  |  |  | PHTF1 | putative homeodomain transcription factor 1 [Source:VGNC Symbol;Acc:VGNC:32848] |
|  |  |  |  |  | MAGI3 | membrane associated guanylate kinase, WW and PDZ domain containing 3 [Source:VGNC Symbol;Acc:VGNC:31148] |
|  |  |  |  |  | LRIG2 | leucine rich repeats and immunoglobulin like domains 2 [Source:VGNC Symbol;Acc:VGNC:30983] |
|  |  |  |  |  | SLC16A1 | solute carrier family 16 member 1 [Source:VGNC Symbol;Acc:VGNC:34683] |
|  |  |  |  |  | TAFA3 | TAFA chemokine like family member 3 [Source:VGNC Symbol;Acc:VGNC:106963] |
|  |  |  |  |  | PPM1J | protein phosphatase, Mg2+/Mn2+ dependent 1J [Source:VGNC Symbol;Acc:VGNC:54471] |
|  |  |  |  |  | RHOC | ras homolog family member C [Source:VGNC Symbol;Acc:VGNC:55672] |
|  |  |  |  |  | MOV10 | Mov10 RNA helicase [Source:VGNC Symbol;Acc:VGNC:31566] |
|  |  |  |  |  | CAPZA1 | capping actin protein of muscle Z-line subunit alpha 1 [Source:VGNC Symbol;Acc:VGNC:26756] |
|  |  |  |  |  | ST7L | suppression of tumorigenicity 7 like [Source:VGNC Symbol;Acc:VGNC:35343] |
|  |  |  |  |  | WNT2B | Wnt family member 2B [Source:VGNC Symbol;Acc:VGNC:36958] |
|  |  |  |  |  | CTTNBP2NL | CTTNBP2 N-terminal like [Source:VGNC Symbol;Acc:VGNC:27823] |
|  |  |  |  |  | KCND3 | potassium voltage-gated channel subfamily D member 3 [Source:VGNC Symbol;Acc:VGNC:30436] |
| Ndama | 5 | 21 | 60500000 | 62300000 | ELK3 | ETS transcription factor ELK3 [Source:VGNC Symbol;Acc:VGNC:28433] |
|  |  |  |  |  | CDK17 | cyclin dependent kinase 17 [Source:VGNC Symbol;Acc:VGNC:27120] |
|  |  |  |  |  | CFAP54 | cilia and flagella associated protein 54 [Source:HGNC Symbol;Acc:HGNC:26456] |
|  |  |  |  |  | NEDD1 | NEDD1 gamma-tubulin ring complex targeting factor [Source:VGNC Symbol;Acc:VGNC:31982] |
|  |  |  |  |  | bta-mir-1251 | bta-mir-1251 [Source:miRBase;Acc:MI0010467] |
|  |  |  |  |  | bta-mir-135a-2 | bta-mir-135a-2 [Source:miRBase;Acc:MI0009736] |
|  | 6 | 18 | 80400000 | 82300000 | EPHA5 | EPH receptor A5 [Source:VGNC Symbol;Acc:VGNC:28535] |
|  | 9 | 12 | 11000000 | 12300000 | RIMS1 | regulating synaptic membrane exocytosis 1 [Source:VGNC Symbol;Acc:VGNC:53801] |
|  |  |  |  |  | KCNQ5 | potassium voltage-gated channel subfamily Q member 5 [Source:VGNC Symbol;Acc:VGNC:53795] |
|  |  |  |  |  | FILIP1 | filamin A interacting protein 1 [Source:VGNC Symbol;Acc:VGNC:29012] |
|  |  |  |  |  | SENP6 | SUMO specific peptidase 6 [Source:VGNC Symbol;Acc:VGNC:52824] |
|  |  |  |  |  | U6 | U6 spliceosomal RNA [Source:RFAM;Acc:RF00026] |
|  |  |  |  |  | MYO6 | myosin VI [Source:VGNC Symbol;Acc:VGNC:31829] |
|  |  |  |  |  | IMPG1 | interphotoreceptor matrix proteoglycan 1 [Source:HGNC Symbol;Acc:HGNC:6055] |
|  |  |  |  |  | HTR1B | 5-hydroxytryptamine receptor 1B [Source:VGNC Symbol;Acc:VGNC:29992] |
|  |  |  |  |  | MEI4 | meiotic double-stranded break formation protein 4 [Source:VGNC Symbol;Acc:VGNC:106818] |
| Zebu-Bororo | 12 | 15 | 28400000 | 30200000 | PDS5B | PDS5 cohesin associated factor B [Source:VGNC Symbol;Acc:VGNC:32718] |
|  |  |  |  |  | N4BP2L2 | NEDD4 binding protein 2 like 2 [Source:VGNC Symbol;Acc:VGNC:53827] |
|  |  |  |  |  | N4BP2L1 | NEDD4 binding protein 2 like 1 [Source:VGNC Symbol;Acc:VGNC:53826] |
|  |  |  |  |  | BRCA2 | BRCA2 DNA repair associated [Source:VGNC Symbol;Acc:VGNC:26554] |
|  |  |  |  |  | ZAR1L | zygote arrest 1 like [Source:VGNC Symbol;Acc:VGNC:107033] |
|  |  |  |  |  | FRY | FRY microtubule binding protein [Source:VGNC Symbol;Acc:VGNC:29122] |
|  |  |  |  |  | RXFP2 | relaxin family peptide receptor 2 [Source:VGNC Symbol;Acc:VGNC:52821] |
|  |  |  |  |  | B3GLCT | beta 3-glucosyltransferase [Source:HGNC Symbol;Acc:HGNC:20207] |
|  |  |  |  |  | bta-mir-2299 | bta-mir-2299 [Source:miRBase;Acc:MI0011309] |
|  |  |  |  |  | HSPH1 | heat shock protein family H (Hsp110) member 1 [Source:VGNC Symbol;Acc:VGNC:53817] |
|  |  |  |  |  | TEX26 | testis expressed 26 [Source:VGNC Symbol;Acc:VGNC:35764] |
|  |  |  |  |  | MEDAG | mesenteric estrogen dependent adiposis [Source:VGNC Symbol;Acc:VGNC:31371] |
|  |  |  |  |  | ALOX5AP | arachidonate 5-lipoxygenase activating protein [Source:VGNC Symbol;Acc:VGNC:25845] |
|  |  |  |  |  | USPL1 | ubiquitin specific peptidase like 1 [Source:VGNC Symbol;Acc:VGNC:36740] |
| Zebu-Fulani | 6 | 28 | 42500000 | 44300000 | PPARGC1A | PPARG coactivator 1 alpha [Source:VGNC Symbol;Acc:VGNC:33184] |
|  |  |  |  |  | DHX15 | DEAH-box helicase 15 [Source:VGNC Symbol;Acc:VGNC:28048] |
